# Cryptic Secondary Metabolites from the Sponge-Associated Fungus *Aspergillus ochraceus*

**DOI:** 10.3390/md17020099

**Published:** 2019-02-03

**Authors:** Marian Frank, Ferhat Can Özkaya, Werner E. G. Müller, Alexandra Hamacher, Matthias U. Kassack, Wenhan Lin, Zhen Liu, Peter Proksch

**Affiliations:** 1 Institute of Pharmaceutical Biology and Biotechnology, Heinrich-Heine-Universität Düsseldorf, 40225 Düsseldorf, Germany; marian.frank@hhu.de; 2 Faculty of Fisheries, İzmir Katip Çelebi University, Çiğli, 35620 İzmir, Turkey; fcanozkaya@gmail.com; 3 Institute of Physiological Chemistry, Universitätsmedizin der Johannes Gutenberg-Universität Mainz, 55128 Mainz, Germany; wmueller@uni-mainz.de; 4 Institute of Pharmaceutical and Medicinal Chemistry, Heinrich-Heine-Universität Düsseldorf, 40225 Düsseldorf, Germany; alexandra.hamacher@hhu.de (A.H.); matthias.kassack@hhu.de (M.U.K.); 5 State Key Laboratory of Natural and Biomimetic Drugs, Peking University, Beijing 100191, China; whlin@bjmu.edu.cn

**Keywords:** Aspergillus ochraceus, OSMAC, co-cultivation, cytotoxicity

## Abstract

The fungus *Aspergillus ochraceus* was isolated from the Mediterranean sponge *Agelas oroides*. The initial fermentation of the fungus on solid rice medium yielded 16 known compounds (**4**–**19**). The addition of several inorganic salts to the rice medium mainly influenced the accumulation of these secondary metabolites. Fermentation of the fungus on white bean medium yielded the new waspergillamide B (**1**) featuring an unusual *p*-nitrobenzoic acid as partial structure. Moreover, two new compounds, ochraspergillic acids A and B (**2** and **3**), which are both adducts of dihydropenicillic acid and *o*- or *p*-aminobenzoic acid, were isolated from the co-culture of the fungus with *Bacillus subtilis*. Compound **2** was also detected in axenic fungal cultures following the addition of either anthranilic acid or tryptophan to the rice medium. The structures of the new compounds were established by 1D and 2DNMR experiments as well as from the HRMS data. The absolute configuration of **1** was elucidated following hydrolysis and derivatization of the amino acids using Marfey’s reagent. Viomellein (**9**) and ochratoxin B (**18**) exhibited strong cytotoxicity against the A2780 human ovarian carcinoma cells with IC_50_ values of 5.0 and 3.0 *µ*M, respectively.

## 1. Introduction

Sponge-associated fungi are known for their production of structurally diverse secondary metabolites, many of which exhibit pharmacological activities such as antibiotic, antiviral, antifungal, and anticancer properties [1,2]. Examples include cephalosporin C which was first isolated and described from the marine-derived fungus *Acremonium chrysogenum* as well as the potential anticancer drug plinabulin which is derived from the fungal metabolitehalimide obtained from a marine *Aspergillus* sp. [3,4]. To date several hundred bioactive compounds have been isolated from marine-derived fungi [5,6]. However, under standard laboratory conditions, fungi will only express a fraction of their biosynthetic potential, whereasthe expression of genes that are not directly involved in growth or differentiation such as those responsible for biosynthesis of natural products are often kept silent to conserve resources [7]. To increase the chances of discovering novel compounds, both the OSMAC (One Strain MAny Compounds) approach and microbial co-cultivation techniques are frequently employed to induce theexpression of silent biogenetic gene clusters [8]. These strategies were also adopted in the present study on *Aspergillus ochraceus*.

*A. ochraceus* was isolated from the inner tissue of the Mediterranean sponge *Agelas oroides*. The fungus was previously reported from the marine environment but is also known as an important food pathogen which is responsible for the production of the carcinogenic mycotoxin ochratoxin A (OTA, **17**) [9,10], as well as of other important mycotoxins such as penicillic acid (PA, **5**) [11], dihydropenicillic acid (DHPA, **6**) [12], and viomellein (**9**) [13]. The toxicological importance of this fungus is highlighted by a disease known as “balcan nephropathy”, which has been linked to the consumption of food products contaminated with PA (**5**) and OTA (**17**) [14,15].

**Figure 1:**
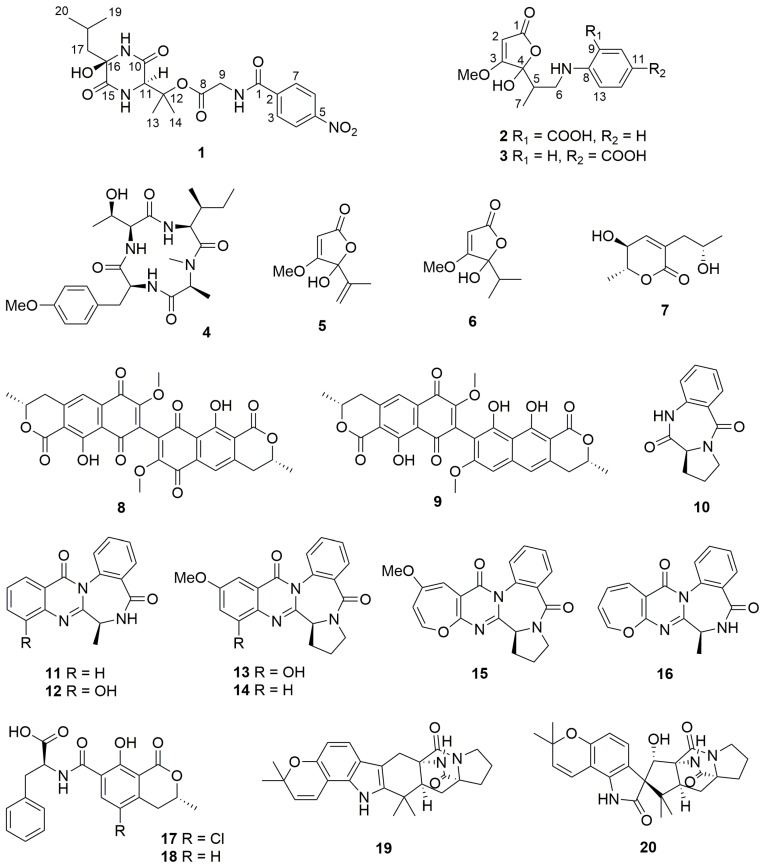
Structures of compounds isolated from *Aspergillusochraceus*.

In this study, the initial fermentation of the marine-derived *A. ochraceus* on solid rice medium containing 3.5% sea salt yielded 16 known metabolites (**4**–**19**). In an attempt to diversify the secondary metabolite pattern, several OSMAC experiments employing different inorganic salts or nitrogen sources were performed. The cultivation of *A. ochraceus* on white beans instead of rice yielded a known alkaloid (**20**) and a new diketopiperazine (**1**), the latter featuring an unusual *p*-nitrobenzoic acid moiety. Moreover, co-cultivation experiments with *Bacillus subtilis* were performed, yielding two new penicillic acid/aminobenzoic acid hybrids (**2** and **3**). Interestingly, the accumulation of **2** could also be provoked by the addition of either anthranilic acid or tryptophan to axenic fungal cultures, suggesting that bacteria are perhaps the source of the aminobenzoic acid moieties of **2** obtained during fungal-bacterial co-cultivation. All isolated metabolites (Figure 1) were tested for their cytotoxicity against the mouse lymphoma cell line L5178Y and the human ovarian carcinoma cell line A2780. Only viomellein (**9**) and ochratoxin B (**18**) exhibited strong cytotoxicity, whereas the other compounds proved to be inactive when assayed at an initial dose of 10 µM.

## 2. Results

The fungus *A. ochraceus* was initially cultivated for 14 days on solid rice medium with the addition of 3.5% artificial sea salt. Chromatographic separation of the EtOAc extract of the fungal culture led to the isolation of several known compounds including violaceotide A (**4**) [16], penicillic acid (**5**) [17], dihydropenicillic acid (**6**) [18], dihydroaspyrone (**7**) [19], xanthomegnin (**8**) [20], viomellein (**9**) [21], cycloanthranilylproline (**10**) [22], circumdatins F (**11**) [23], G (**12**) [23], E (**13**) [24], H (**14**) [25], B (**15**) [26], and L (**16**) [27], ochratoxins A (**17**) [28] and B (**18**) [29], and stephacidin A (**19**) [30].

Different cultivation experiments with organic or inorganic supplements were performed in order to diversify the metabolite pattern. The addition of inorganic salts to the rice medium mainly influenced the accumulation of these secondary metabolites causing either an increase or a decrease in their concentrations of compounds when compared to the cultivation of the fungus on solid rice medium containing sea salt (Table 1).

When the fungus was cultivated on white beans instead of rice (both media containing 3.5% NaCl), the alkaloid concentrations (**11**, **12**, and **15**–**18**) remained high in the former extract, whereas those of all non-alkaloid compounds (**5**–**9**) were strongly decreased. This made the investigation of the alkaloid fraction easier, resulting in the isolation of a new diketopiperazine, waspergillamide B (**1**), and a known alkaloid, sclerotiamide (**20**) [31] (Table 2).

**Figure 2:**
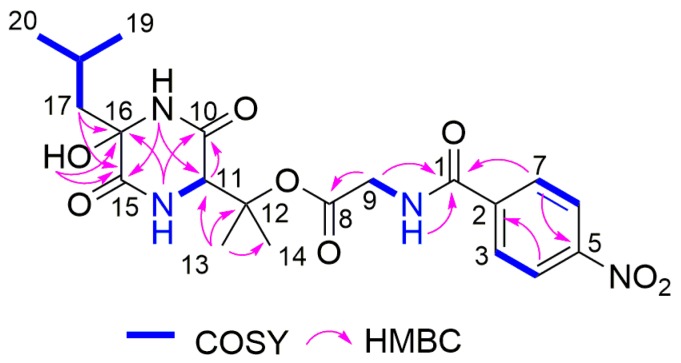
COSY and key HMBC correlations of **1**.

Comparison of the ^1^H and ^13^C NMR data (Table 3) between **1** and waspergillamide A isolated from an Australian mud dauber wasp-associated *Aspergillus* sp. [32] suggested the replacement of the trisubstituted double bond at C-16/C-17 by a methylene group (*δ*_C_ 47.2, *δ*_H_ 2.01, and 1.54) and an oxygenated quaternary carbon (*δ*_C_ 80.5 and signal of a hydroxy group at *δ*_H_ 6.24) in **1**. This is further supported by the molecular formula of **1** (C_20_H_26_N_4_O_8_) containing an additional H_2_O molecule compared to waspergillamide A as evident from the HRESIMS data of **1**. The COSY correlations between H_ab_-17 and H-18 as well as the HMBC correlation from H_ab_-17 and 16-OH to C-15 and C-16 confirmed the location of a methylene group at C-17 and the attachment of a hydroxy group at C-16 in **1**. The remaining substructure of **1** was identical to that of waspergillamide A on the basis of detailed analysis of the 2D NMR spectra of **1** (Figure 2 and Figures S1–S8). The diketopiperazine moiety in **1**is formed from 3-hydroxyvaline and 2-hydroxyleucine residues, the latter being related to theΔ^2,3^-leucine residue present in waspergillamide A [32]. To assign the absolute configuration of the 3-OH-Val residue in **1**, acid hydrolysis products of **1** were derivatized with both enantiomers of Marfey’s reagent (D- and L-FDAA). It has been reported that 3-OH-D-Val-D-FDAA eluted prior to 3-OH-D-Val-L-FDAA in HPLC [32,33]. In our study, the product with D-FDAA eluted at 3.95 min, whereas the adduct with L-FDAA eluted at 4.72 min, indicating the presence of a 3-OH-D-Val residue in **1**. In addition, the NOE correlation from 16-OH to Me-14 suggested that these protons were on the same face of the diketopiperazine ring, which allowed the assignment of the 16*R* configuration for **1**. Thus, the structure of **1**, for which the name waspergillamide B is proposed, was elucidated as shown.

Co-cultivation experiments of *A. ochraceus* with *Streptomyces lividans* caused an increase in the concentrations of PA (**5**) and DHPA (**6**), whereas the co-cultivation of *A. ochraceus* with *B. subtilis* yielded two new penicillic acid derivatives (**2** and **3**) that were absent in axenic fungal or bacterial controls (Table 4).

**Figure 3:**
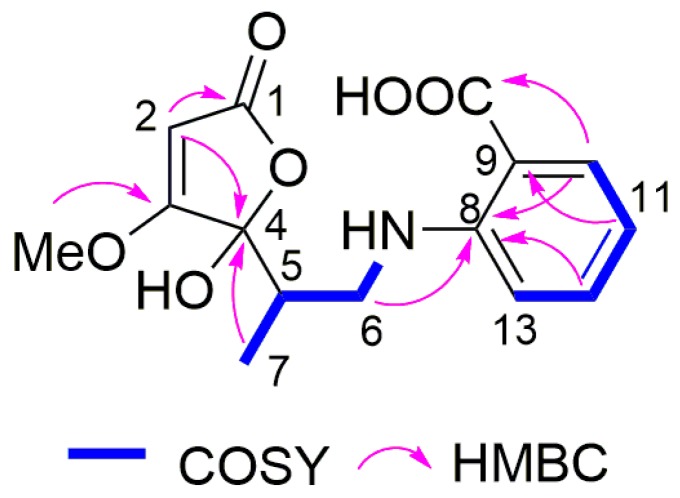
COSY and key HMBC correlations of **2**.

Compound **2** was isolated as yellowish oil. Based on the HRESIMS data, the molecular formula was established as C_15_H_17_NO_6_. The ^1^HNMR spectrum of **2** (Table 5) exhibited four mutually coupled aromatic protons at *δ*_H_ 7.88 (dd, H-10), 6.56 (br t, H-11), 7.35 (ddd, H-12), and 6.79 (br d, H-13), suggesting the presence of an *o*-disubstituted benzene ring. An *o*-aminobenzoic acid moiety was established by the COSY correlations between H-10/H-11/H-12/H-13 in addition to the HMBC correlations from H-10 to C-8 at *δ*_C_ 152.2 and a carboxy carbon at *δ*_C_ 171.1, from H-11 to C-9 at *δ*_C_ 111.3, and from H-12 to C-8 (Figure 3 and Figures S9–S14). The remaining NMR data of **2** (Table 5) were compatible with those of co-isolated dihydropenicillic acid (**6**). A dihydropenicillic acid moiety was established by the COSY correlations between H_ab_-6/H-5/Me-7 together with the HMBC correlations from H-2 (*δ*_H_ 5.24) to C-1 (*δ*_C_ 172.7) and C-4 (*δ*_C_ 106.2), from the protons of the methoxy group (*δ*_H_ 3.94) to C-3 (*δ*_C_ 181.6), and from Me-7 (*δ*_H_ 0.96) to C-4. Moreover, the HMBC correlation from H_ab_-6 to C-8 confirmed the linkage between dihydropenicillic acid moiety and *o*-aminobenzoic acid moiety through an amine bond. Thus, the structure of **2** was elucidated as shown. The trivial name ochraspergillic acid A is suggested for this compound. Duplicated signals with a ratio around 5 to 4 observed in the NMR spectra of **2** were caused by tautomerism at C-4, which is common for penicillic acid derivatives [34].

Ochraspergillic acid B (**3**) shared the same molecular formula with **2** on the basis of the HRESIMS data. The ^1^HNMR data of **3** (Table 6) were similar to those of **2** and also exhibited two sets of signals with a ratio of 4:3. However, signals of a *p*-disubstituted benzene ring at *δ*_H_7.78 (d, H-10/12) and 6.62 (d, H-9/13) were observed for **3**. The HMBC correlation from H-9/13 to C-11 (*δ*_C_ 118.0) and from H-10/12 to C-8 (*δ*_C_ 153.9) and a carboxy carbon (*δ*_C_ 170.4) confirmed the presence of a *p*-aminobenzoic acid moiety in **3**. Detailed analysis of the 2D NMR data of **3** (Figures S15–S20) revealed that the remaining substructure of **3** was identical to that of **2**.

Most of the identified alkaloids are biogenetically derived from anthranilic acid or from tryptophan. Interestingly, compounds **2** and **3** were only detected during co-cultivation of the fungus with *B. subtilis* but not during co-cultivation with *S. lividans*. Thus, the fungus responds to the presence of different bacteria by the accumulation of different metabolites. Similar results were recently reported for the co-cultivation of the fungus *Chaetomium* sp. with *Pseudomonas aeruginosa* [35,36] compared to a co-culture of the fungus with *B. subtilis* [37]. Compounds **2** and **3** seem to be biotransformation products of penicillic acid (**5**). Thus, a feeding experiment was performed by adding either anthranilic acid or l-tryptophan to solid rice medium (Table 7). Compound **2** could be unequivocally detected following the addition of either 1% or 2% of anthranilic acid or of 2% tryptophan to rice medium. Moreover, the concentration of **2** increased in cultures growing in the presence of 2% anthranilic acid compared to 1% anthranilic acid. These results suggest that anthranilic acid or tryptophan which are biogenetic building blocks in the formation of **2** and **3** during fungal-bacterial co-cultivation may possibly betraced back to bacterial instead of fungal metabolism as the fungus fails to produce these compounds in the absence of added precursors.

All isolated compounds were subjected to a cytotoxicity assay against the L5178Y mouse lymphoma cell line and against the A2780 human ovarian carcinoma cell line. Viomellein (**9**) and ochratoxin B (**18**) exhibited strong cytotoxicity against the human ovarian carcinoma cell line A2780 with IC_50_ values of 5.0 and 3.0 *µ*M, respectively. In addition, viomellein (**9**) showed significant cytotoxicity against the mouse lymphoma cell line L5178Y with an IC_50_ value of 5.3 *µ*M. Viomellein (**9**) was further tested against the human Jurkat T and Ramos B cell lines but was shown to be inactive against the latter two cell lines.It is worth mentioning that viomellein (**9**) and ochratoxins A (**17**) and B (**18**) are well-known mycotoxins. Viomellein (**9**) has been reported to induce mycotoxicosis in mice [38], whereas ochratoxins A (**17**) and B (**18**) have been associated with several human and animal diseases including poultry ochratoxicosis, porcine nephropathy, and human endemic nephropathies [39]. Compared to **18**, the substitution of proton by chlorine in **17**decreased its cytotoxicity against A2780 cells (a growth inhibition of 90% when **17** was tested at 100 μM).

## 3. Discussion and Conclusions

In conclusion, this study on the sponge-derived fungus *A. ochraceus* yielded twenty compounds, which can be classified into peptides (**4**, **17**, **18**), diketopiperazine alkaloids (**1**, **19**, **20**), penicillic acid derivatives (**2**, **3**, **5**, and **6**), polyketides (**7**–**9**) and benzodiazepine alkaloids (**10**–**16**). The majority of compounds was biogenetically derived from anthranilic acid or tryptophan. The cultivation of the fungus on protein rich media (white beans) yielded the new waspergillamide B (**1**), whereas the co-cultivation of *A. ochraceus* with *B. subtilis* yielded two new compounds, ochraspergillic acids A and B (**2** and **3**). The latter two compounds seem to be biogenetically derived from both fungal and bacterial metabolism as indicated by precursor feeding using anthranilic acid or tryptophan.

## 4. Materials and Methods

### 4.1. General Experimental Procedures

Specific optical rotations were measured using a P-1020 polarimeter (JASCO, Tokyo, Japan). High-resolution mass spectra were measured with a UHR-QTOF maXis 4G (Bruker Daltonics, Bremen, Germany) mass spectrometer, whereas low-resolution mass spectra were measured with a Finnigan LCQ Deca (Thermo, Bremen, Germany) mass spectrometer. 1D and 2D NMR spectra were acquired using Bruker Avance III 300 or 600 (Bruker, Karlsruhe, Germany) spectrometers. Analytical HPLC chromatograms were obtained using a DIONEX 3000 system coupled to an Ultimate 3000 diode array UV detector (Thermo Scientific, Germering, Germany) and a Eurospher C18 (Knauer, Berlin, Germany) separation column (125 × 4 mm i.d., 5 *μ*m). HPLC analysis for Marfey’s reaction was performed on a KNAUER AZURAsystem using a Knauer Smartline UV Detector 2600 (Knauer, Berlin, Germany) and a EC250/4.6 NUCLEOSIL 120-5 C4 (Macherey-Nagel, Dueren, Germany) separation column. Column chromatography was performed using Sephadex LH-20 (Sigma-Aldrich, Steinheim, Germany) or silica gel 60M as stationary phases. Thin layer chromatography (TLC) was performed using pre-coated silica gel 60 F254 plates (Merck, Darmstadt, Germany) with detection under 254 and 365 nm followed by anisaldehyde spray reagent. Semi-preparative HPLC purification was performed using a Lachrom-Merck Hitachi (Merck, Darmstadt, Germany) system (L7100 pump and L7400 UV detector) with a KNAUER Eurospher C18 column (300 × 8 mm i.d., 10 μm).

### 4.2. Fungal Material

The sponge-associated fungus was isolated from the tissue of the marine sponge *Agelas oroides*, which was collected at a depth of 10 m in Sığaçık-İzmir, Turkey. The fungal strain was identified as *Aspergillus ochraceus* (GenBank accession number MK168605) by amplification and sequencing of the ITS-Region including the 5.8S ribosomal DNA and subsequent BLAST search as previously described [40].

### 4.3. Fermentation and Extraction

For the initial fermentation, 10 1 L Erlenmeyer flasks were filled with solid rice medium (100 g rice from Oryza Milchreis, 3.8 g of sea salt, and 110 mL of demineralized water each) and autoclaved at 121 °C for 20 min. After cooling to room temperature, the flasks were inoculated, each with a 2–4 cm^2^ piece of agar plate, on which the fungus had been growing for one week. The fermentation was continued under static conditions for 14 days at 20 °C. At that time, the fungus had completely overgrown the medium. Fermentation was terminated by the addition of 350 mL of EtOAc. After soaking in EtOAc overnight, the mycelium was cut into pieces with a spatula and shaken continuously for 8 h at 150 rpm, after which the extract was collected via filtration through a paper filter. The residue was washed with 50 mL of EtOAc, filtered, and evaporated to dryness, yielding 17.4 g of an oily substance. A liquid–liquid partitioning was performed between MeOH-H_2_O (90:10) and *n*-hexane. After evaporation to dryness, the *n*-hexane phase weight amounted to 13.0 g and the methanolic phase weight amounted to 4.3 g. After HPLC analysis of both phases, only the methanolic phase was further chromatographically investigated.

#### 4.3.1. OSMAC Experiment

To investigate the influences of several inorganic salts or organic supplements to the growth media, the cultivation of the fungus was performed on 100 g of solid rice medium with the addition of 110 mL of demineralized water and either 3.5% NaCl, 3.5% NaBr, 3.5% NaI, 1% NaNO_3_, 1% NaNO_2_, 1% NH_4_Cl, 1% peptone, or 1% yeast extract. The controls consisted of 100 g of solid rice medium with 3.5% sea salt and 110 mL of demineralized water. After autoclaving, the media were inoculated with ~2 cm^2^ of overgrown agar plate. The fungus grew on all media at similar growth rates. The experiment was terminated by adding 400 mL of EtOAc to each flask, after which the medium was soaked overnight, cut into pieces, and shaken for 8 h before filtration. After evaporation of EtOAc, the extract was dissolved in 50 mL MeOH and analyzed by HPLC-DAD. Two inoculated flasks were used per experiment. The experiment with white bean medium was conducted using five 100 g flasks of white beans, which were soaked overnight in demineralized water prior to autoclaving.

#### 4.3.2. Co-Cultivation Experiment

Three flasks containing 100 g of autoclaved rice each were inoculated with 10 mL of a bacterial broth (optical density 0.2) and pre-incubated for 4 days at 40°C (*B. subtilis*) or 25°C (*S. lividans*), respectively, before the fungus was introduced. Co-cultivation was performed at 25°C for an additional 14 days. The resulting EtOAc extracts were chromatographically compared to the axenic control cultures of the fungus and the bacteria, which were grown under the same conditions. The experiment was repeated in an identical manner except for an extended pre-incubation period of the bacteria for 14 days.

#### 4.3.3. Feeding Experiment

Cultures were grown on two flasks containing 100 g of solid rice medium each, which was spiked with 1% or 2% of either anthranilic acid (neutralized with 1 M NaOH at pH = 7) or l-tryptophan (set to pH = 10 with 1M NaOH for solubility) before autoclaving. Fungal control cultures were grown under the same conditions and the EtOAc extracts were chromatographically compared.

### 4.4. Isolation of Compounds

The crude extract of the initial large-scale fermentation was subjected to vacuum liquid chromatography (VLC) using silica gel as stationary phase and a gradient of solvent mixtures. The solvent gradient consisted of *n*-hexane-EtOAc (10:0, 8:2, 6:4, 4:6, 2:8), CH_2_Cl_2_-MeOH (10:0, 90:10, 80:20, 70:30, 60:40, 50:50, 40:60, 30:70, 20:80), and an additional wash step of MeOH with 0.1 % TFA. The resulting 15 fractions (V1 to V15) were analyzed by HPLC. Fractions V7 and V8 were combined (3.1 g) and subjected to another VLC on silica gel using a gradient of EtOAc-DCM (30:70, 40:60, 50:50, 60:40, 70:30, 80:20, 90:10, 100:0) initially and then EtOAc-MeOH (90:10, 85:15, 80:20, 75:25, 70:30, 40:60, 10:90) to yield 14 subfractions (V7/8-V1 to V7/8-V14). Fractions V7/8-V3 and V7/8-V4 were combined amounting to 298 mg and subjected to a Sephadex LH-20 column using acetone as mobile phase to yield four subfractions (V7/8-V3/4-SD1 to V7/8-V3/4-SD4). Fraction V7/8-V3/4-SD3 was separated by a silica gel column using CH_2_Cl_2_-MeOH (98:2) as mobile phase followed by purification using semi- preparative HPLC with a gradient of MeOH-H_2_O (55% 0–3 min, 55% to 90% 3–23 min, and 90% 23–25 min), yielding **14** (1.1 mg) and **16** (1.6 mg). Fraction V7/8-V3/4-SD4 was further purified by semi-preparative HPLC with a gradient of MeOH-H_2_O (50% 0–3 min, 50% to 65% 3–18 min, and 100% 18–28 min), yielding **19** (0.7 mg), **13** (0.8 mg), **11** (3.8 mg), and **12** (21.4 mg).Fractions V9 and V10 were combined (282 mg) and further purified by a Sephadex LH-20 column using acetone as mobile phase to give **17** (62.9 mg).

The extract (1.8 g) of the 1% NH_4_Cl culture was separated by VLC on silica gel as described above to give 14 fractions (V1 to V14). Fraction V4 was subsequently purified on a Sephadex LH-20 column using acetone as eluent to yield **5** (79.8 mg). Fraction V6 was suspended in acetone and centrifuged three times to give **4** (10.0 mg).

The extract (7.5 g) of 1% NaNO_2_ culture was also fractionated by VLC on silica gel as described for the crude extract of the initial large-scale fermentation. Fraction V6 (125 mg) was separated on a Sephadex LH-20 column using MeOH as eluent and subsequently purified by semi-preparative HPLC with a gradient of MeOH-H_2_O from 20% to 100% over 25 minutes to yield **7** (10.6 mg).

The EtOAc extract (4.4 g) of the bean culture was subjected to a silica gel VLC column as described above. Fraction V5 was further purified by a silica gel column to yield **15** (33.0 mg) and **16** (61.0 mg). After separation on a Sephadex LH-20 column and subsequent purification by semi-preparative HPLC, fraction V6 yielded **1** (1.6 mg) and **20** (2.6 mg), whereas fraction V9 yielded **17** (1.0 mg) and **18** (1.1 mg).

The EtOAc extract obtained from the co-culture of the fungus with *B. subtilis* was partitioned between 300 mL *n*-hexane and 400 mL of 90% MeOH-H_2_O to give 3.2 g of oily *n*-hexane soluble extract, 4.1 g of methanol soluble dry extract, and 193 mg of an insoluble fraction. The insoluble fraction was dissolved in CH_2_Cl_2_ and subjected to a Sephadex LH-20 column followed by separation using semi-preparative HPLC with a gradient of CH_3_CN-H_2_O from 40% to 80% over 20 minutes to yield **9** (2.5 mg) and **8** (3.1 mg). The MeOH soluble fraction was fractionated by a silica gel VLC as described above. Fraction V3 was chromatographed over a Sephadex LH-20 column and subsequently separated by semi-preparative HPLC to yield **5** (80.0 mg) and **6** (59.0 mg). Fraction V5 yielded **7** (24.3 mg), **2** (1.5 mg), **3** (1.3 mg), **10** (3.3 mg), **13** (2.9 mg), **12** (3.7 mg), and **11** (0.4 mg) following a similar procedure as for fraction V3. Fraction V6 was purified on a Sephadex LH-20 column using acetone as eluent to give **4** (15.0 mg).

Waspergillamide B (**1**): white needles; [α]_D_^23^ +22 (c 0.2, MeOH); UV (MeOH) *λ*_max_ 267 nm; ^1^H and ^13^C NMR data, Table 3; HRESIMS *m/z* 451.1824 [M + H]^+^ (calcd for C_20_H_27_N_4_O_8_, 451.1828).

Ochraspergillic acid A (**2**): yellow oil; UV (MeOH) *λ*_max_ 353, 254, and 221 nm; ^1^H and ^13^C NMR data, Table 5; HRESIMS *m/z* 308.1134 [M + H]^+^ (calcd for C_15_H_18_NO_6_, 308.1129).

Ochraspergillic acid B (**3**): yellow oil; UV (MeOH) *λ*_max_ 306 and 224 nm; ^1^H and ^13^C NMR data, Table 6; HRESIMS *m/z* 308.1127 [M + H]^+^ (calcd for C_15_H_18_NO_6_, 308.1129).

### 4.5. Marfey’s Reaction for **1**

The general procedure was adapted from the C3-method [32,33]. Two aliquots of 100 µg of **1** were hydrolyzed in 200 µL of 6M HCl at 110°C for 12 h in a closed glass vial. Subsequently, HCl was removed by drying under a nitrogen stream. The dry residues were treated with 20 µL of 1M aqueous NaHCO_3_. Next, 100 µL of a 1% acetone solution of L-FDAA (1-fluoro-2, 4-dinitrophenyl-5-L-alanine amide) were added to the first aliquot of **1**, while 100 µL of a 1% acetone solution of D-FDAA were added to the second aliquot of **1**. The mixtures were kept at 40°C for 1 h and occasionally shaken by hand. To stop the reaction, 20 µL of 1M HCl were added. The mixtures were centrifuged at 13,000 rpm for 10 min and an aliquot of 10 µL was analyzed using an RP-HPLC column with UV detection at 340 nm. The retention times of the L- and D-FDAA product of **1** were compared to each other. A Macherey-Nagel EC250/4.6 NUCLEOSIL250-5 C4-column (4 × 300 mm, i.d.) was used for HPLC analysis. The gradient of MeOH-H_2_O was changed from 15% to 60% MeOH over 55 min at a flow rate of 1 mL/min (Figures S21–S22).

### 4.6. Cytotoxicity Assay

Cytoxocicity was investigated using the MTT (3-(4,5-dimethylthiazol-2-yl)-2,5-diphenyltetrazolium bromide) method against the mouse lymphoma cell line L5178Y or the human ovarian carcinoma cell line A2780 as previously described [41,42]. As positive controls, kahalalide F for L5178Y (IC_50_ 4.3 µM) and cisplatin for A2780 (IC_50_ 2.2 µM) were used. As negative controls,0.1% ethylene glycol monomethyl ether in DMSO was used for L5178Y, whereas0.9% NaCl, 0.1% DMSO, and 1% DMSO were used for A2780.

## Figures and Tables

**Table 1:** Relative concentrations of selected compounds (in mAU*min at 235 nm) in cultures of *A. ochraceus* grown on solid rice medium (control) vs. cultures grown in the presence of different inorganic salts (*n* = 2 in each case, fermentation for 14 days).

Compounds	Control	3.5% NaCl	3.5% NaBr	3.5% NaI	1% NH_4_Cl	1% NaNO_3_	1% NaNO_2_
**4**	28.8	29.4	42.0	21.6	22.2	61.3	28.1
**5**	576.7	257.1	633.1	314.1	447.6	149.0	152.6
**6**	242.6	28.7	83.7	360.5	20.7	122.0	69.1
**7**	115.6	38.7	30.0	61.5	11.8	23.6	33.8
**8**	97.9	81.7	21.0	76.9	n.d.	163.2	23.6
**9**	112.1	116.8	633.1	63.9	28.8	329.0	152.6
**11**	23.4	51.8	77.3	8.6	128.0	51.1	5.3
**12**	8.7	49.7	69.8	9.7	39.6	66.6	12.6
**13**	n.d. ^a^	24.9	n. d.	n.d.	n.d.	n.d.	n.d.
**14**	n.d.	n.d.	92.7	n.d.	148.5	n.d.	n.d.
**15**	158.1	296.5	450.4	40.4	527.7	224.2	17.8
**16**	34.5	154.9	179.5	22.1	80.6	83.1	13.7
**17**	275.6	237.1	30.0	59.5	25.9	180.4	33.8
**18**	105.8	11.4	144.8	n.d.	n.d.	n.d.	5.5
**19**	123.8	140.3	190.4	14.6	174.6	116.9	5.4

^a^ n.d. = not detected in the HPLC chromatogram of the crude extract.

**Table 2:** Relative concentrations of selected compounds (in mAU*min at 235 nm) in cultures of *A. ochraceus* grown on solid rice medium (control) vs. cultures grown on white bean medium (*n* = 5 in each case, fermentation for 14 days).

Compound	Control	Beans
**1**	n.d.	10.4
**4**	18.1	16.4
**5**	767.8	n.d.
**6**	4.7	n.d.
**7**	18.3	n.d.
**8**	103.1	n.d.
**9**	175.5	11.9
**11**	12.8	18.2
**12**	2.3	73.9
**15**	104.3	105.7
**16**	16.5	68.2
**17**	84.7	57.4
**18**	n.d. ^a^	18.5
**19**	24.8	3.5
**20**	n.d.	3.5

^a^ n.d. = not detected in the HPLC chromatogram of the crude extract.

**Table 3:** ^1^H and ^13^C NMR data for **1**. ^a^

No.	*δ*_C_, type	*δ*_H_ (*J* in Hz)
1	165.1, C	
2	139.3, C	
3,7	128.7, CH	8.10, d (8.9)
4,6	123.5, CH	8.34, d (8.9)
5	149.1, C	
8	168.6, C	
9	42.3, CH2	4.03, d (5.8)
9-NH		9.21, t (5.8)
10	164.3, C	
11	60.5, CH	4.34, d (2.8)
11-NH		8.17, d (2.8)
12	83.6, C	
13	23.7, CH3	1.60, s
14	22.3, CH3	1.43, s
15	167.6, C	
16	80.5, C	
16-NH		8.73, s
16-OH		6.24, s
17	47.2, CH2	2.01, m
		1.54, m
18	23.9, CH	1.55, m
19	23.4, CH3	0.87, d (6.5)
20	22.5, CH3	0.79, d (6.5)

^a^ Measured in DMSO-*d*_6_ (^1^H at 600 MHz and ^13^C at 150 MHz).

**Table 4:** Relative concentrations of selected compounds (in mAU*min at 235 nm) in cultures of *A. ochraceus* grown on solid rice medium (control) vs. co-cultures (fermentation for 14 days) with *Streptomyceslividans* or *Bacillus subtilis* after 4 days (*n* = 3) or 14 days (*n* = 2) of bacterial pre-incubation. ^a^

Compound	*A. ochraceus* Control 4+14 days	*A. ochraceus* + *S. lividans* 4+14 days	*A. ochraceus* + *B. subtilis* 4+14 days	*A. ochraceus* Control 14+14 days	*A. ochraceus* + *S. lividans* 14+14 days	*A. ochraceus* + *B. subtilis* 14+14 days
**2**	n.d. ^b^	n.d.	0.4	n.d.	n.d.	36.2
**4**	19.2	21.3	60.4	n.d.	n.d.	n.d.
**5**	350.7	215.1	213.7	452.1	2128.7	745.8
**6**	691.6	144.8	144.1	349.5	1015.2	268.3
**7**	131.5	18.1	63.1	56.3	105.6	122.7
**8**	n.d.	n.d.	n.d.	379.9	193.9	102.2
**9**	1.9	0.4	0.4	406.3	164.4	110.8
**11**	16.1	17.0	13.5	125.8	54.8	60.4
**12**	19.6	19.2	18.5	44.6	16.7	n.d.
**15**	8.6	13.2	13.3	826.7	273.9	339.7
**16**	4.0	2.5	1.9	118.8	67.0	39.5
**17**	37.6	16.7	18.0	28.9	9.6	23.6
**19**	n.d.	n.d.	n.d.	707.9	239.4	209.7

^a^ Compound **3** overlaps other compounds in the HPLC chromatogram and thus is not quantifiable. ^b^ n.d. = not detected in the HPLC chromatogram of the crude extract.

**Table 5:** ^1^H and ^13^C NMR data for **2**. ^a^

No.	2 (Ma) ^b^		2 (Mi) ^b^	
	*δ*_C_, type ^c^	*δ*_H_ (*J* in Hz)	*δ*_C_, type ^c^	*δ*_H_ (*J* in Hz)
1	172.7, C		173.0, C	
2	89.9, CH	5.24, s	89.9, CH	5.20, s
3	181.6, C		181.7, C	
4	106.2, C		105.7, C	
5	38.8, CH	2.33, m	39.8, CH	2.38, m
6	43.8, CH_2_	3.75, dd (13.4, 3.9)	44.8, CH_2_	3.35, m
		3.09, dd (13.4, 8.8)		3.06, dd (13.7, 7.1)
7	12.6, CH_3_	0.96, d (6.9)	12.3, CH_3_	1.13, d (6.9)
8	152.2, C		152.2, C	
9	111.3, C		111.1, C	
10	133.0, CH	7.88, dd (7.7, 1.8)	133.0, CH	7.88, dd (7.7, 1.8)
11	115.3, CH	6.56, br t (7.7)	115.3, CH	6.56, br t (7.7)
12	135.5, CH	7.35, ddd (8.4, 7.7, 1.8)	135.5, CH	7.34, ddd (8.4, 7.7, 1.8)
13	111.9, CH	6.79, br d (8.4)	111.8, CH	6.67, br d (8.4)
3-OMe	60.3, CH_3_	3.94, s	60.2, CH_3_	3.88, s
9-COOH	171.7, C		171.7, C	

^a^ Measured in CD_3_OD (^1^H at 600 MHz and ^13^C at 150 MHz). ^b^ Ma and Mi denote the major and minor epimers, respectively. ^c^ Data extracted from the HSQC and HMBC spectra.

**Table 6:** ^1^H and ^13^C NMR data for **3**. ^a^

No.	3 (Ma) ^b^		3 (Mi) ^b^	
	*δ*_C_, type ^c^	*δ*_H_ (*J* in Hz)	*δ*_C_, type ^c^	*δ*_H_ (*J* in Hz)
1	172.7, C		173.0, C	
2	90.0, CH	5.26, s	90.1, CH	5.22, s
3	181.4, C		181.3, C	
4	106.2, C		105.7, C	
5	38.4, CH	2.32, m	39.8, CH	2.37, m
6	44.3, CH_2_	3.71, dd (13.6, 3.8)	44.7, CH_2_	3.32, m
		3.03, dd (13.6, 9.0)		2.98, dd (13.7, 8.2)
7	12.8, CH_3_	0.91, d (6.9)	12.1, CH_3_	1.10, d (6.9)
8	153.9, C		153.9, C	
9,13	112.2, CH	6.62, d (8.8)	112.1, CH	6.55, d (8.8)
10,12	132.6, CH	7.78, d (8.8)	132.6, CH	7.77, d (8.8)
11	118.0, C		118.4, C	
3-OMe	60.3, CH_3_	3.94, s	60.3, CH_3_	3.92, s
11-COOH	170.4, C		170.4, C	

^a^ Measured in CD_3_OD (^1^H at 600 MHz and ^13^C at 150 MHz). ^b^ Ma and Mi denote the major and minor epimers, respectively. ^c^ Data extracted from the HSQC and HMBC spectra.

**Table 7:** Relative concentrations of selected compounds (in mAU*min at 235 nm) in cultures of *A. ochraceus* grown on solid rice medium with pH = 7 or 10 (control) vs. cultures grown in the presence of 1% or 2% anthranilic acid or l-tryptophan (*n* = 2 in each case, fermentation for 14 days). ^a^

Compound	Control pH = 7	Anthranilic acid 1%	Anthranilic acid 2%	Control pH = 10	Tryptophan 1%	Tryptophan 2%
**2**	n.d. ^b^	15.4	61.4	n.d.	n.d.	31.6
**4**	10.8	6.5	n.d.	5.5	10.6	8.9
**5**	244.1	242.1	90.7	303.3	178.7	145.2
**6**	77.2	76.8	38.6	92.5	126.7	161.6
**7**	9.8	4.2	3.2	5.3	11.5	11.0
**8**	5.0	14.7	26.2	3.7	3.9	24.2
**9**	44.6	3.5	79.7	28.7	34.8	59.6
**11**	8.4	1.3	3.3	3.2	5.4	7.0
**12**	5.9	5.6	7.8	4.3	9.1	12.8
**15**	6.1	7.9	n.d.	9.1	9.0	n.d.
**16**	3.1	3.3	n.d.	2.1	3.2	n.d.
**17**	44.5	46.3	36.6	36.4	43.4	35.4
**18**	3.6	2.6	3.7	n.d.	12.2	4.5

^a^ Compound **3** overlaps other compounds in the HPLC chromatogram and thus is not quantifiable. ^b^ n.d. = not detected in the HPLC chromatogram of the crude extract.

## Supplementary Materials

The following are available online at http://www.mdpi.com/1660-3397/17/2/99/s1, UV, HRMS, 1D and 2D NMR spectra of all the new compounds **1**–**3** as well as chromatograms for Marfey’s products of **1**.
