# Supplementary material for: Cryptic Secondary Metabolites from the Sponge-Associated Fungus Aspergillus ochraceus
Source: Mar Drugs. 2019 Feb 3;17(2):99. doi: 10.3390/md17020099 (PMC6410417; doi:10.3390/md17020099)

Supplementary Material

Cryptic Secondary Metabolites from the Sponge-Associated Fungus *Aspergillus ochraceus*

Marian Frank [1], Ferhat Can Özkaya [2], Werner E. G. Müller [3], Alexandra Hamacher [4], Matthias U. Kassack [4], Wenhan Lin [5], Zhen Liu [1,*] and Peter Proksch [1,*]

[1] Institute of Pharmaceutical Biology and Biotechnology, Heinrich-Heine-Universität Düsseldorf, 40225 Düsseldorf, Germany; marian.frank@hhu.de (M.F.)
[2] Faculty of Fisheries, İzmir Katip Çelebi University, Çiğli, 35620 İzmir, Turkey; fcanozkaya@gmail.com (F.C.Ö.)
[3] Institute of Physiological Chemistry, Universitätsmedizin der Johannes Gutenberg-Universität Mainz, 55128 Mainz, Germany; wmueller@uni-mainz.de (W.E.G.M.)
[4] Institute of Pharmaceutical and Medicinal Chemistry, Heinrich-Heine-Universität Düsseldorf, 40225 Düsseldorf, Germany; alexandra.hamacher@hhu.de (A.H.); matthias.kassack@hhu.de (M.U.K.)
[5] State Key Laboratory of Natural and Biomimetic Drugs, Peking University, Beijing 100191, People's Republic of China; whlin@bjmu.edu.cn (W.L.)
* Correspondence: zhenfeizi0@sina.com (Z.L.); proksch@uni-duesseldorf (P.P.); Tel.: +49-211-81-14163

Table of Content

Figure S1. UV spectrum of compound **1**. 3

Figure S2. HRESIMS of compound **1**. 3

Figure S3. ^{1}HNMR (600 MHz, DMSO-d_6) spectrum of compound **1**. 4

Figure S4. ^{13}C NMR (125 MHz, DMSO-d_6) spectrum of compound **1**. 5

Figure S5. COSY (600MHz, DMSO-d_6) spectrum of compound **1.** 6

Figure S6. HSQC (600MHz/150 MHz, DMSO-d_6) spectrum of compound **1**. 7

Figure S7. HMBC (600MHz/150 MHz, DMSO-d_6) spectrum of compound **1**. 8

Figure S8. ROESY (600MHz, DMSO-d_6) spectrum of compound **1**. 9

Figure S9. UV spectrum of compound **2**. 10

Figure S10. HRESIMS of compound **2**. 10

Figure S11. ^{1}H NMR (600 MHz, MeOH-d_4) spectrum of compound **2**. 11

Figure S12. COSY (600 MHz, MeOH-d_4) spectrum of compound **2**. 12

Figure S13. HSQC (600MHz/150 MHz, MeOH-d_4) spectrum of compound **2**. 13

Figure S14. HMBC (600MHz/150 MHz, MeOH-d_4) spectrum of compound **2**. 14

Figure S15. UV spectrum of compound **3**. 15

Figure S16. HRESIMS of compound **3**. 15

Figure S17. ^{1}H NMR (600 MHz, MeOH-d_4) spectrum of compound **3**. 16

Figure S18. COSY (600 MHz, MeOH-d_4) spectrum of compound **3**. 17

Figure S19. HSQC (600MHz/150 MHz, MeOH-d_4) spectrum of compound **3**. 18

Figure S20. HMBC (600MHz/150 MHz, MeOH-d_4) spectrum of compound **3**. 19

Figure S21. Chromatogram of C_4-Marfey's L-FDAA adduct of compound **1**. 20

Figure S22. Chromatogram of C_4-Marfey's D-FDAA adduct of compound **1**. 21

Figure S1. UV spectrum of compound **1**.

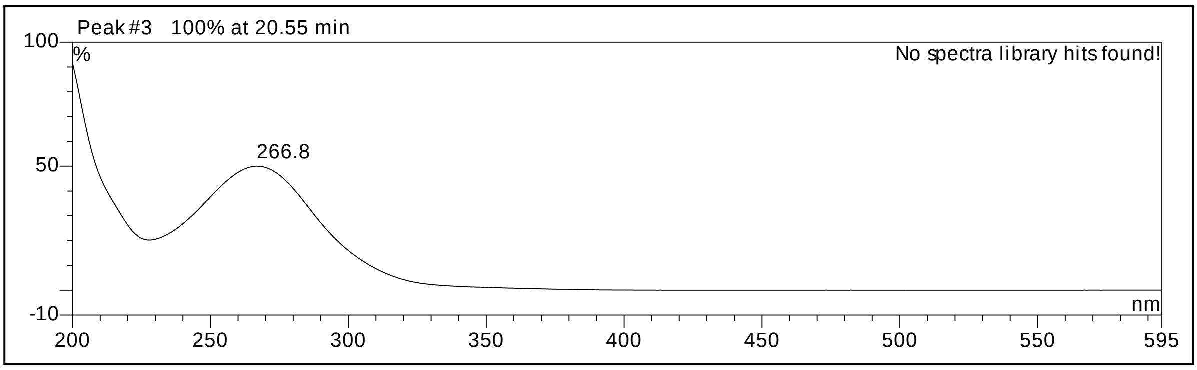

Figure S2. HRESIMS of compound **1**.

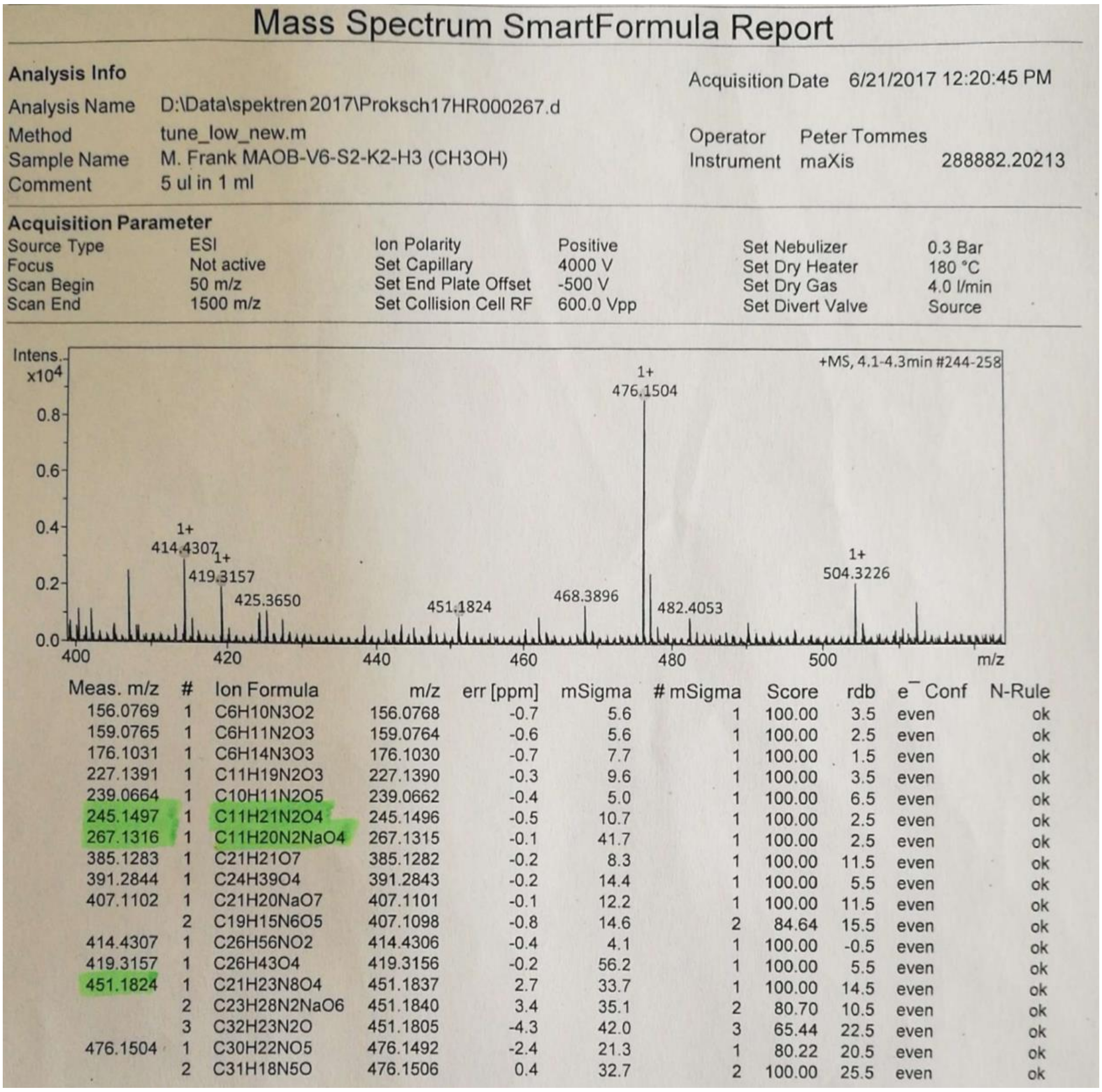

Mass Spectrum SmartFormula Report

Analysis Info

Analysis Name D:\Data\spektren 2017\Proksch17HR000267.d
Method tune_low_new.m
Sample Name M. Frank MAOB-V6-S2-K2-H3 (CH3OH)
Comment 5 ul in 1 ml

Acquisition Date 6/21/2017 12:20:45 PM
Operator Peter Tommes
Instrument maXis 288882.20213

Acquisition Parameter

Source Type	ESI	Ion Polarity	Positive	Set Nebulizer	0.3 Bar
Focus	Not active	Set Capillary	4000 V	Set Dry Heater	180 °C
Scan Begin	50 m/z	Set End Plate Offset	-500 V	Set Dry Gas	4.0 l/min
Scan End	1500 m/z	Set Collision Cell RF	600.0 Vpp	Set Divert Valve	Source

Meas. m/z	#	Ion Formula	m/z	err [ppm]	mSigma	# mSigma	Score	rdb	e⁻ Conf	N-Rule
156.0769	1	C6H10N3O2	156.0768	-0.7	5.6	1	100.00	3.5	even	ok
159.0765	1	C6H11N2O3	159.0764	-0.6	5.6	1	100.00	2.5	even	ok
176.1031	1	C6H14N3O3	176.1030	-0.7	7.7	1	100.00	1.5	even	ok
227.1391	1	C11H19N2O3	227.1390	-0.3	9.6	1	100.00	3.5	even	ok
239.0664	1	C10H11N2O5	239.0662	-0.4	5.0	1	100.00	6.5	even	ok
245.1497	1	C11H21N2O4	245.1496	-0.5	10.7	1	100.00	2.5	even	ok
267.1316	1	C11H20N2NaO4	267.1315	-0.1	41.7	1	100.00	2.5	even	ok
385.1283	1	C21H21O7	385.1282	-0.2	8.3	1	100.00	11.5	even	ok
391.2844	1	C24H39O4	391.2843	-0.2	14.4	1	100.00	5.5	even	ok
407.1102	1	C21H20NaO7	407.1101	-0.1	12.2	1	100.00	11.5	even	ok
	2	C19H15N6O5	407.1098	-0.8	14.6	2	84.64	15.5	even	ok
414.4307	1	C26H56NO2	414.4306	-0.4	4.1	1	100.00	-0.5	even	ok
419.3157	1	C26H43O4	419.3156	-0.2	56.2	1	100.00	5.5	even	ok
451.1824	1	C21H23N8O4	451.1837	2.7	33.7	1	100.00	14.5	even	ok
	2	C23H28N2NaO6	451.1840	3.4	35.1	2	80.70	10.5	even	ok
	3	C32H23N2O	451.1805	-4.3	42.0	3	65.44	22.5	even	ok
476.1504	1	C30H22NO5	476.1492	-2.4	21.3	1	80.22	20.5	even	ok
	2	C31H18N5O	476.1506	0.4	32.7	2	100.00	25.5	even	ok

Figure S3. ^{1}HNMR (600 MHz, DMSO-d_6) spectrum of compound **1**.

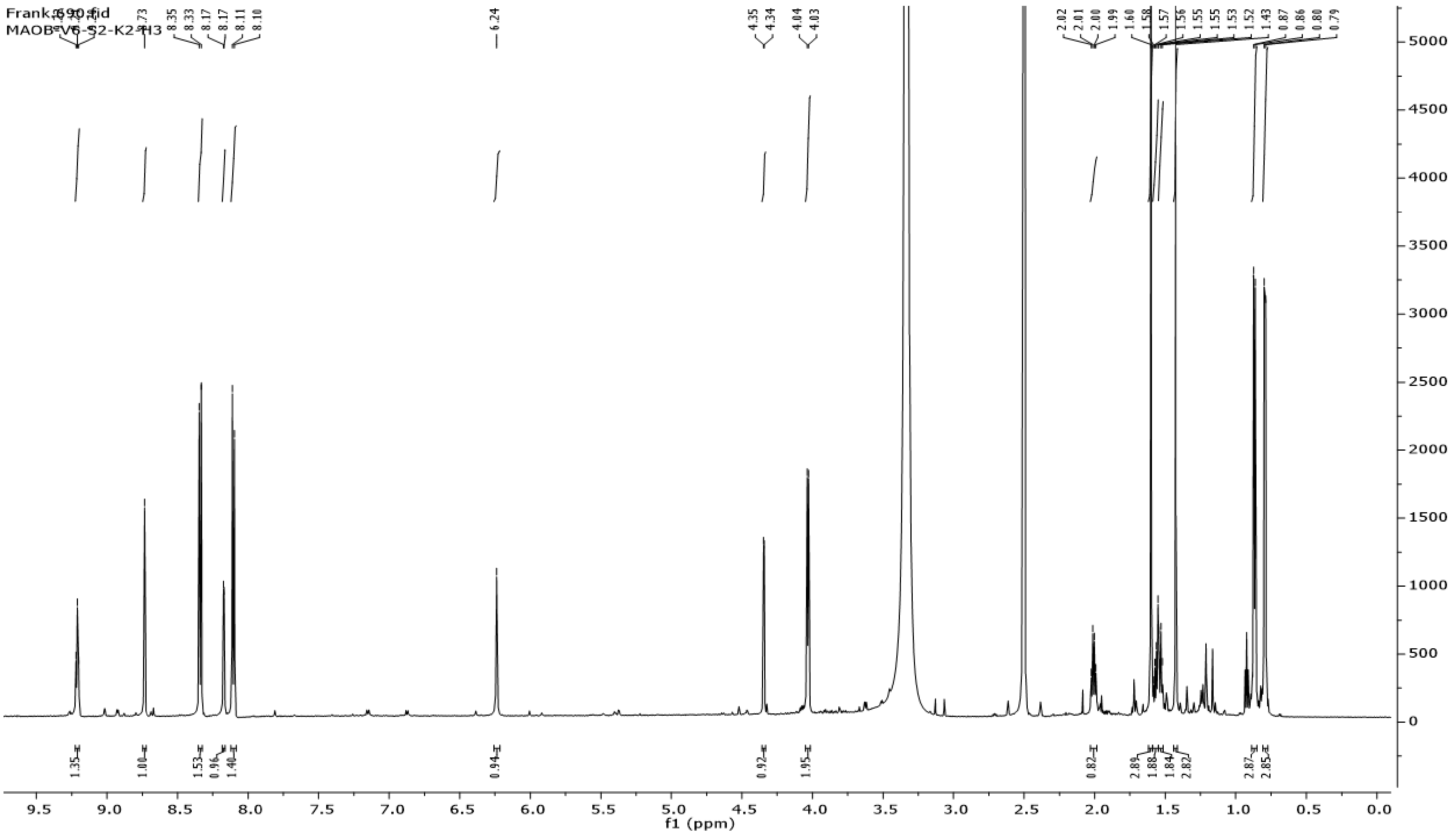

Figure S4. ^{13}C NMR (125 MHz, DMSO-d_6) spectrum of compound **1**.

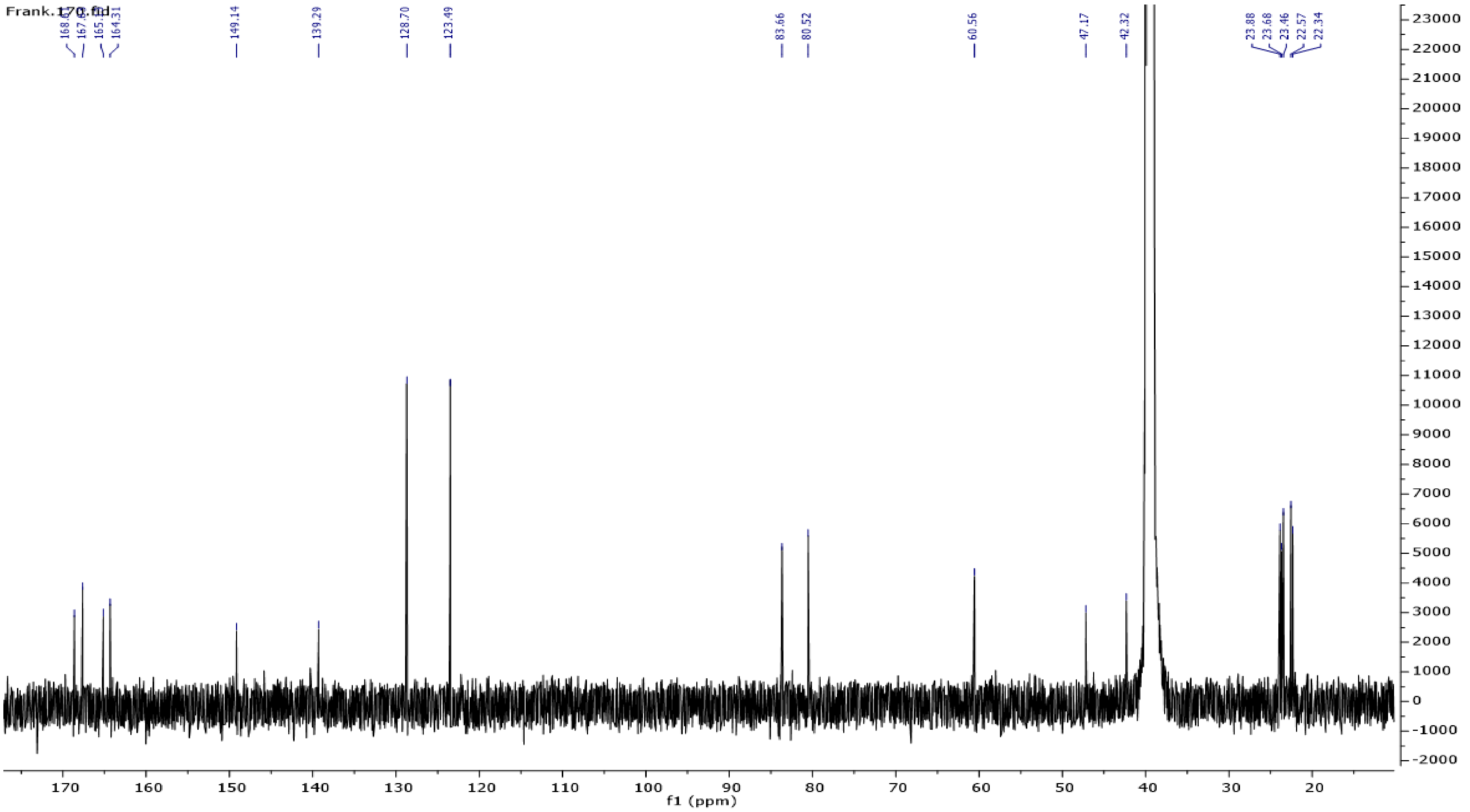

Figure S5. COSY (600MHz, DMSO-d_6) spectrum of compound **1.**

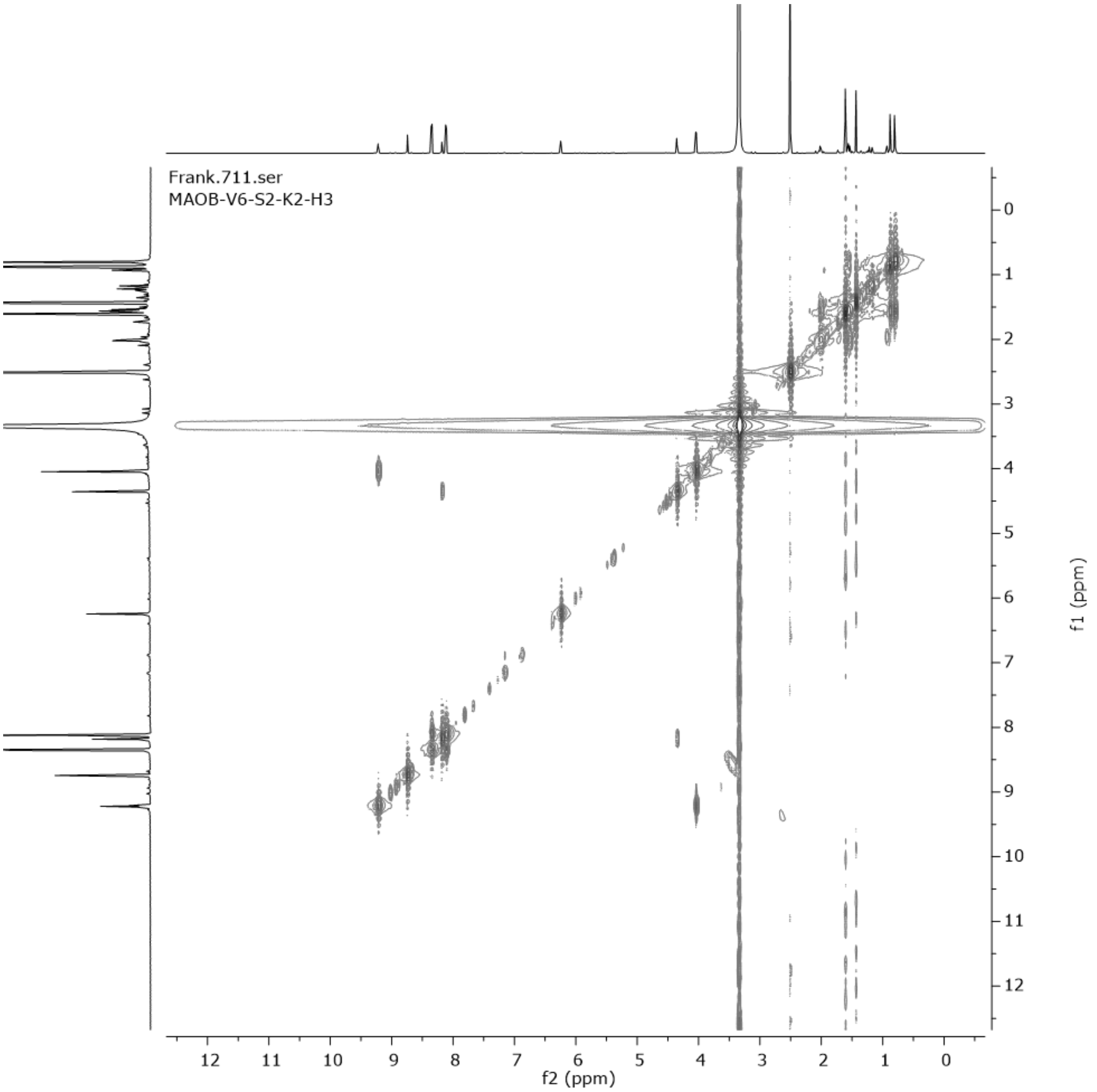

Figure S6. HSQC (600MHz/150 MHz, DMSO-d_6) spectrum of compound **1**.

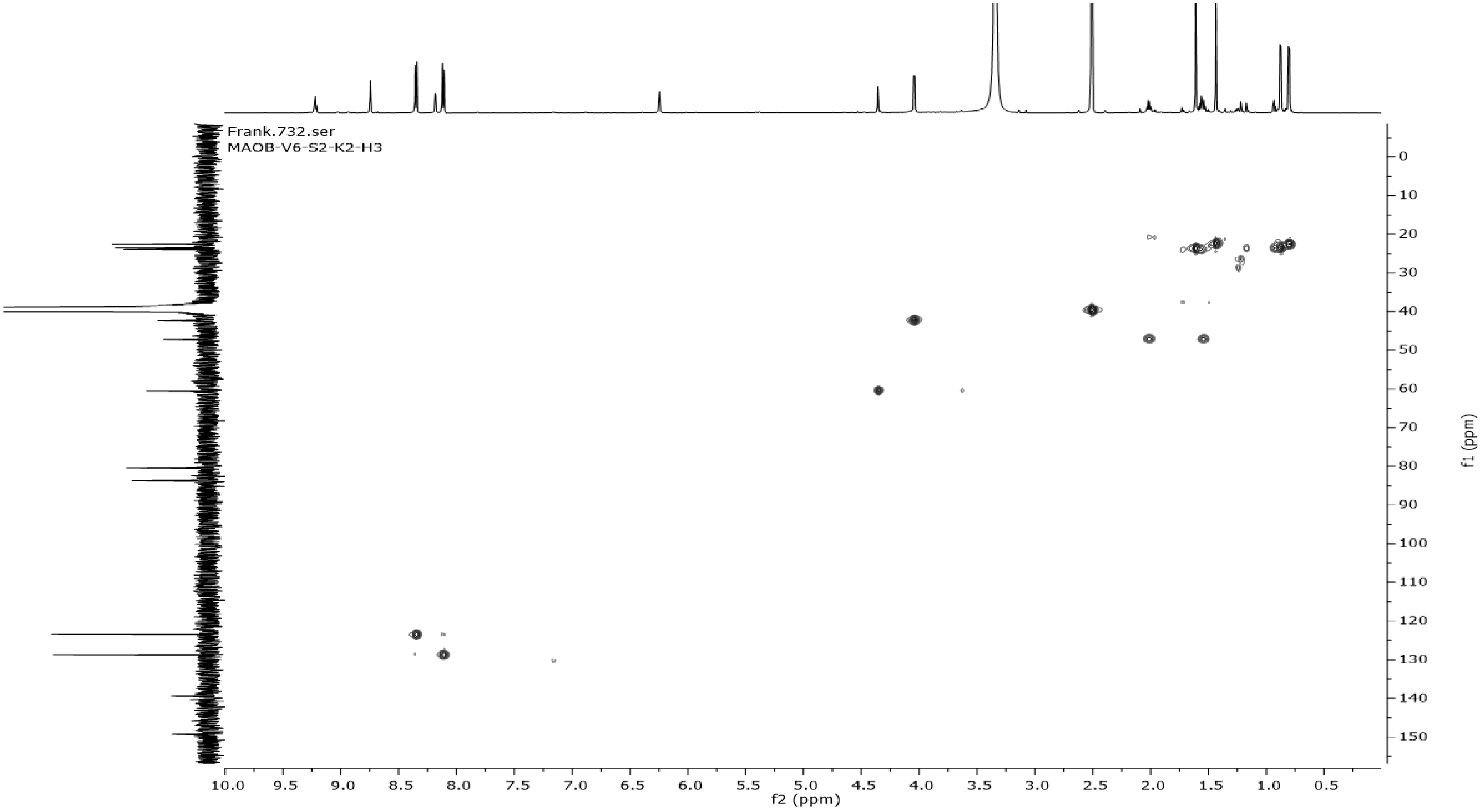

Figure S7. HMBC (600MHz/150 MHz, DMSO-d_6) spectrum of compound **1**.

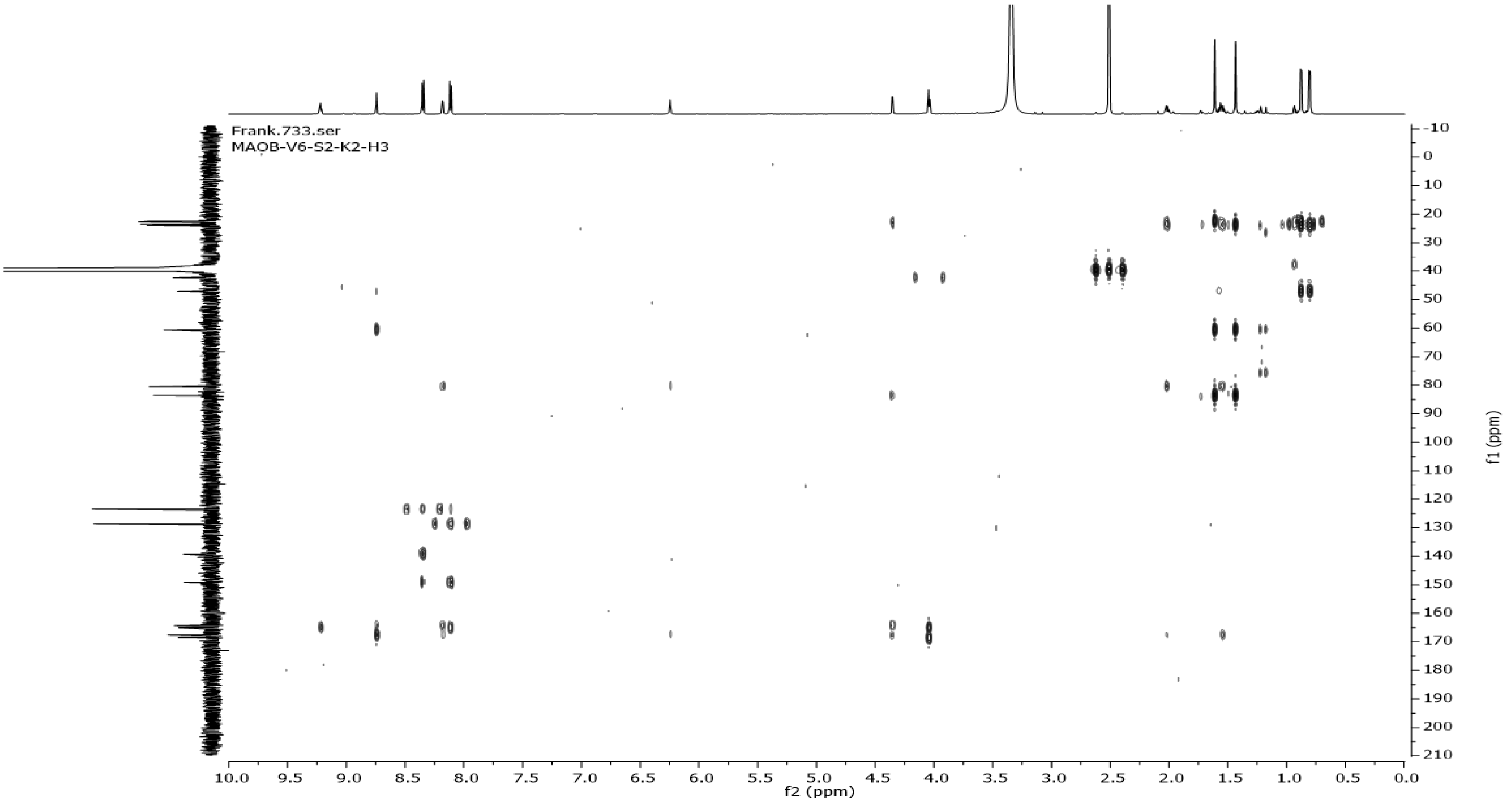

Figure S8. ROESY (600MHz, DMSO-d_6) spectrum of compound **1**.

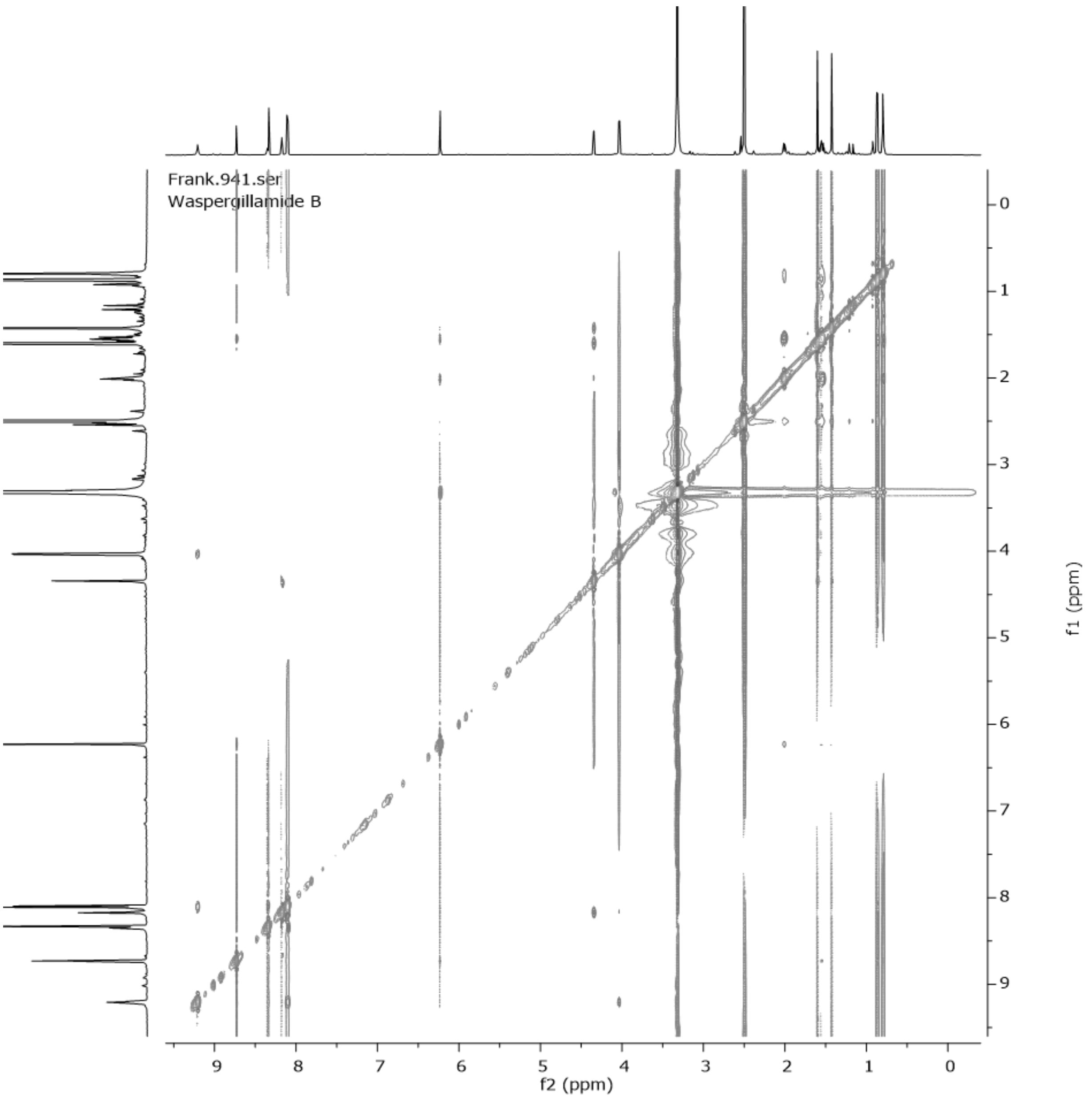

Figure S9. UV spectrum of compound **2**.

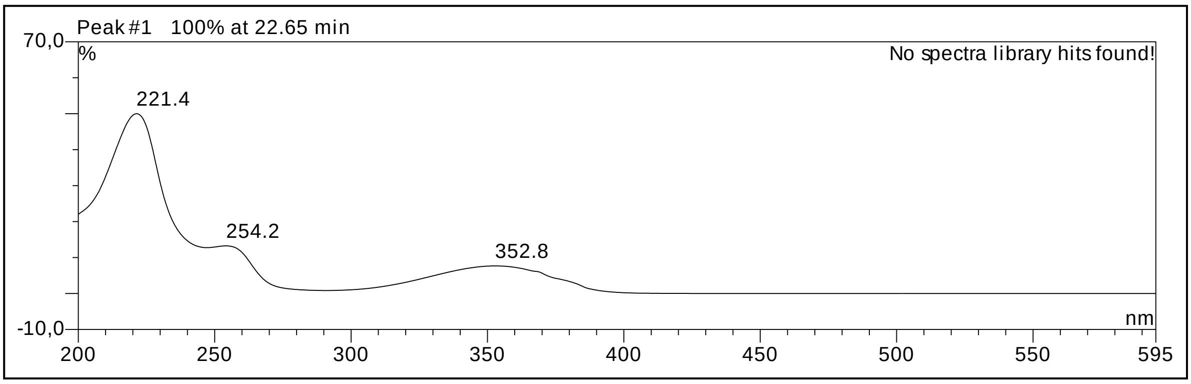

Figure S10. HRESIMS of compound **2**.

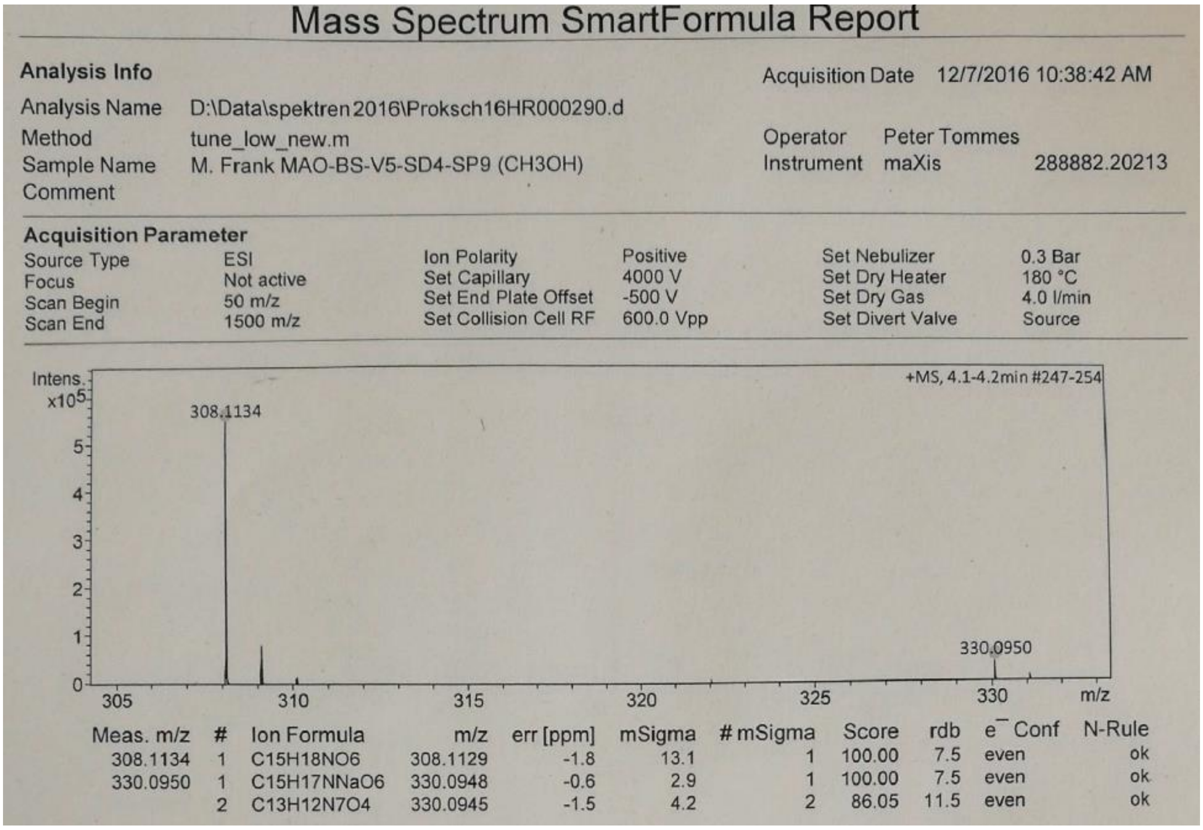

Mass Spectrum SmartFormula Report

Analysis Info

Analysis Name	D:\Data\spektren 2016\Proksch16HR000290.d	Acquisition Date	12/7/2016 10:38:42 AM
Method	tune_low_new.m	Operator	Peter Tommes
Sample Name	M. Frank MAO-BS-V5-SD4-SP9 (CH3OH)	Instrument	maXis 288882.20213
Comment			

Acquisition Parameter

Source Type	ESI	Ion Polarity	Positive	Set Nebulizer	0.3 Bar
Focus	Not active	Set Capillary	4000 V	Set Dry Heater	180 °C
Scan Begin	50 m/z	Set End Plate Offset	-500 V	Set Dry Gas	4.0 l/min
Scan End	1500 m/z	Set Collision Cell RF	600.0 Vpp	Set Divert Valve	Source

Meas. m/z	#	Ion Formula	m/z	err [ppm]	mSigma	# mSigma	Score	rdb	e⁻ Conf	N-Rule
308.1134	1	C15H18NO6	308.1129	-1.8	13.1	1	100.00	7.5	even	ok
330.0950	1	C15H17NNaO6	330.0948	-0.6	2.9	1	100.00	7.5	even	ok
	2	C13H12N7O4	330.0945	-1.5	4.2	2	86.05	11.5	even	ok

Figure S11. ^{1}H NMR (600 MHz, MeOH-d_4) spectrum of compound **2**.

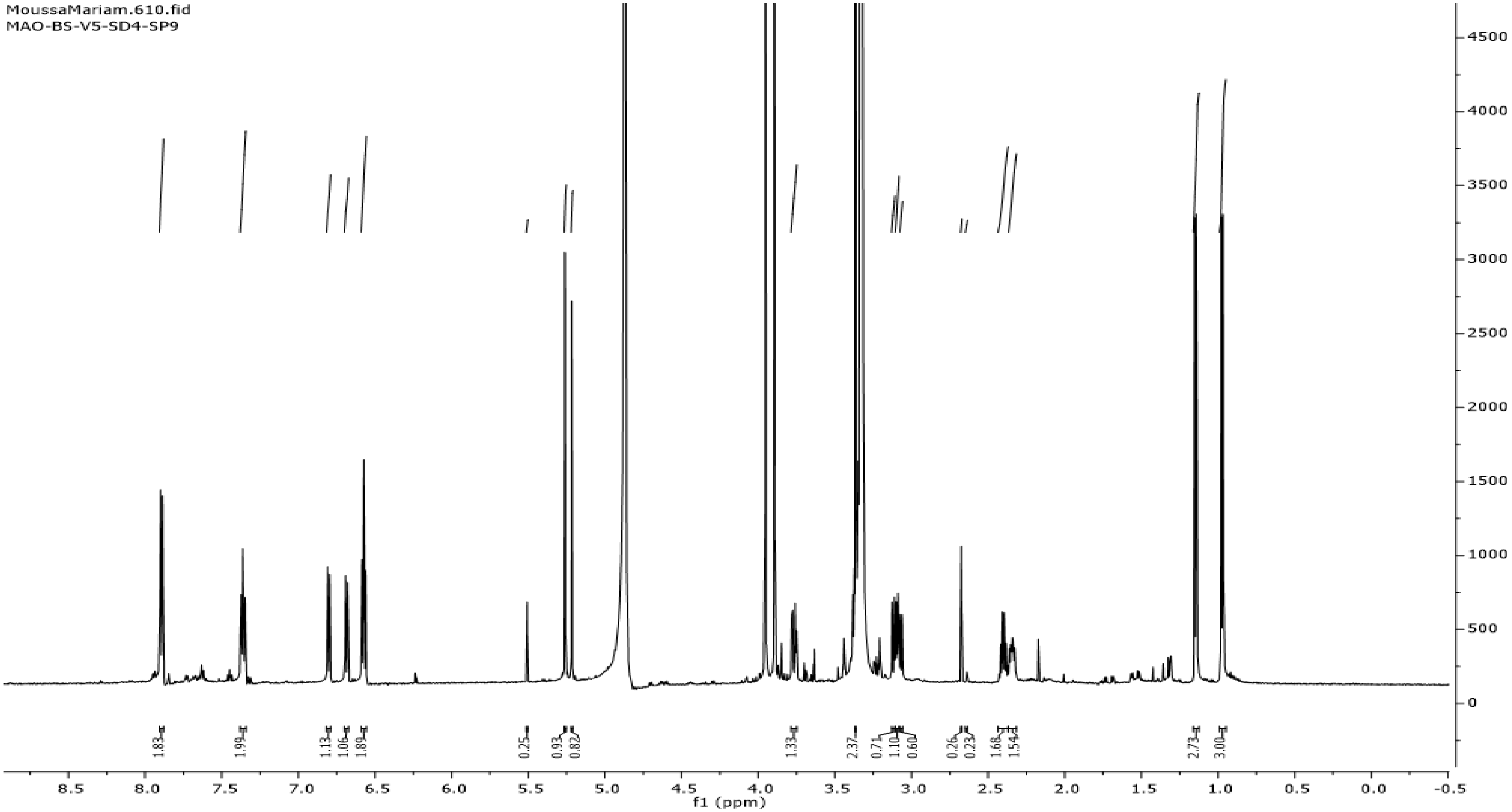

Figure S12. COSY (600 MHz, MeOH-d_4) spectrum of compound **2**.

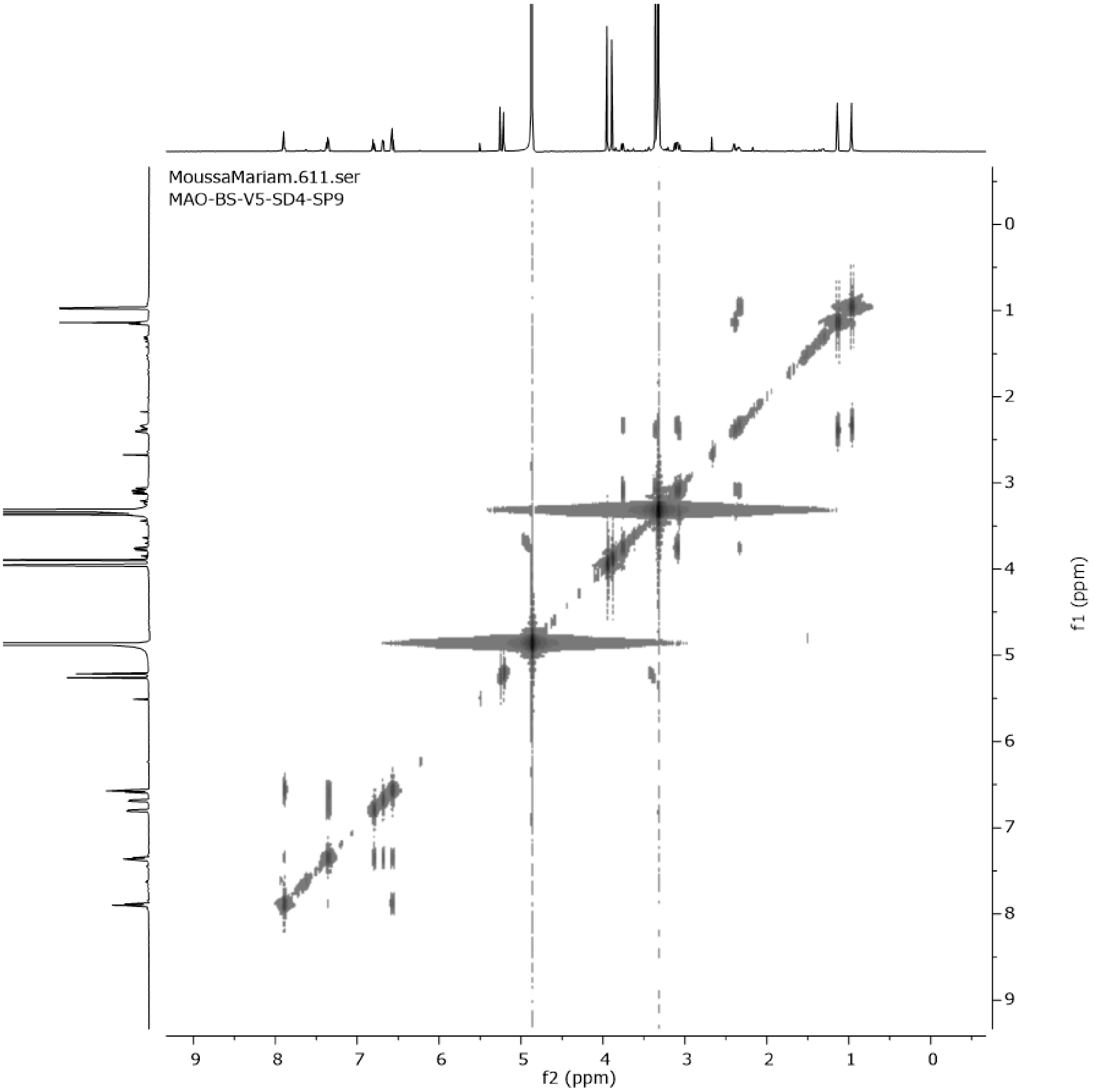

Figure S13. HSQC (600MHz/150 MHz, MeOH-d_4) spectrum of compound **2**.

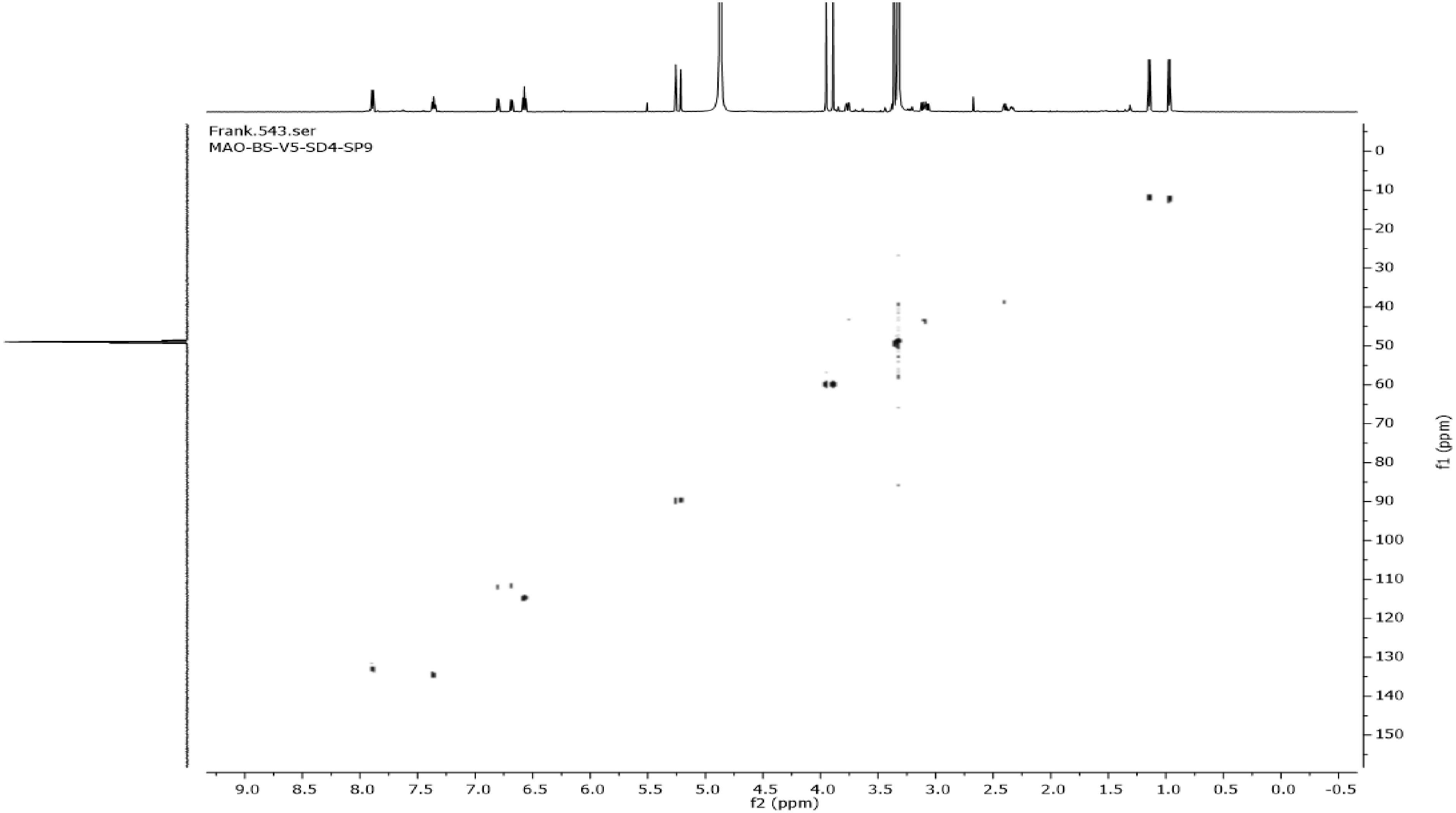

Figure S14. HMBC (600MHz/150 MHz, MeOH-d_4) spectrum of compound **2**.

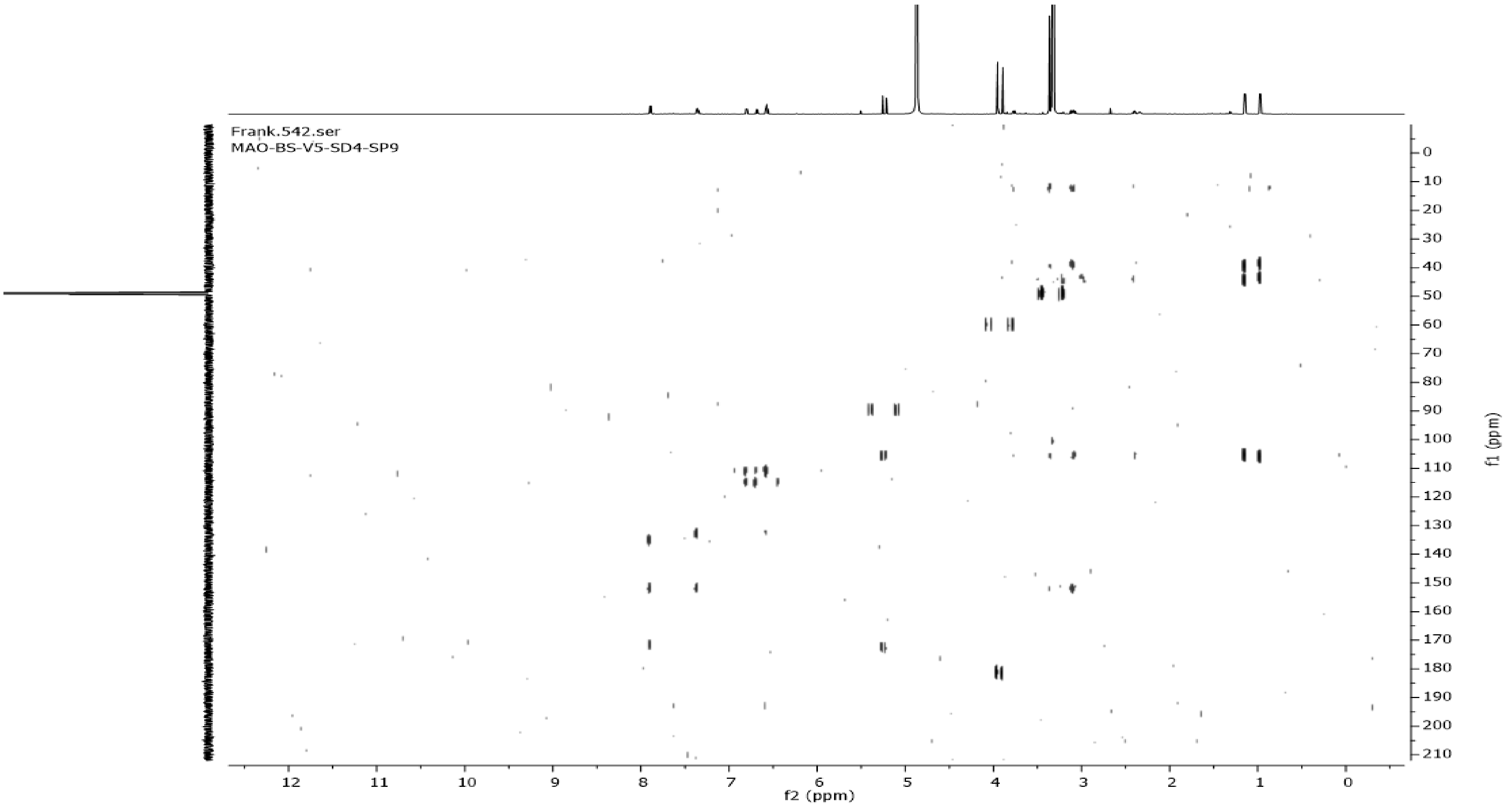

Figure S15. UV spectrum of compound **3**.

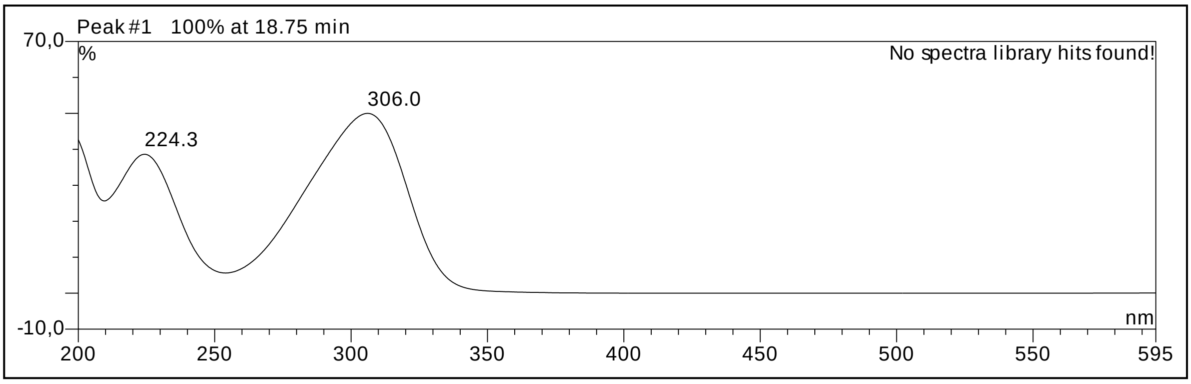

Figure S16. HRESIMS of compound **3**.

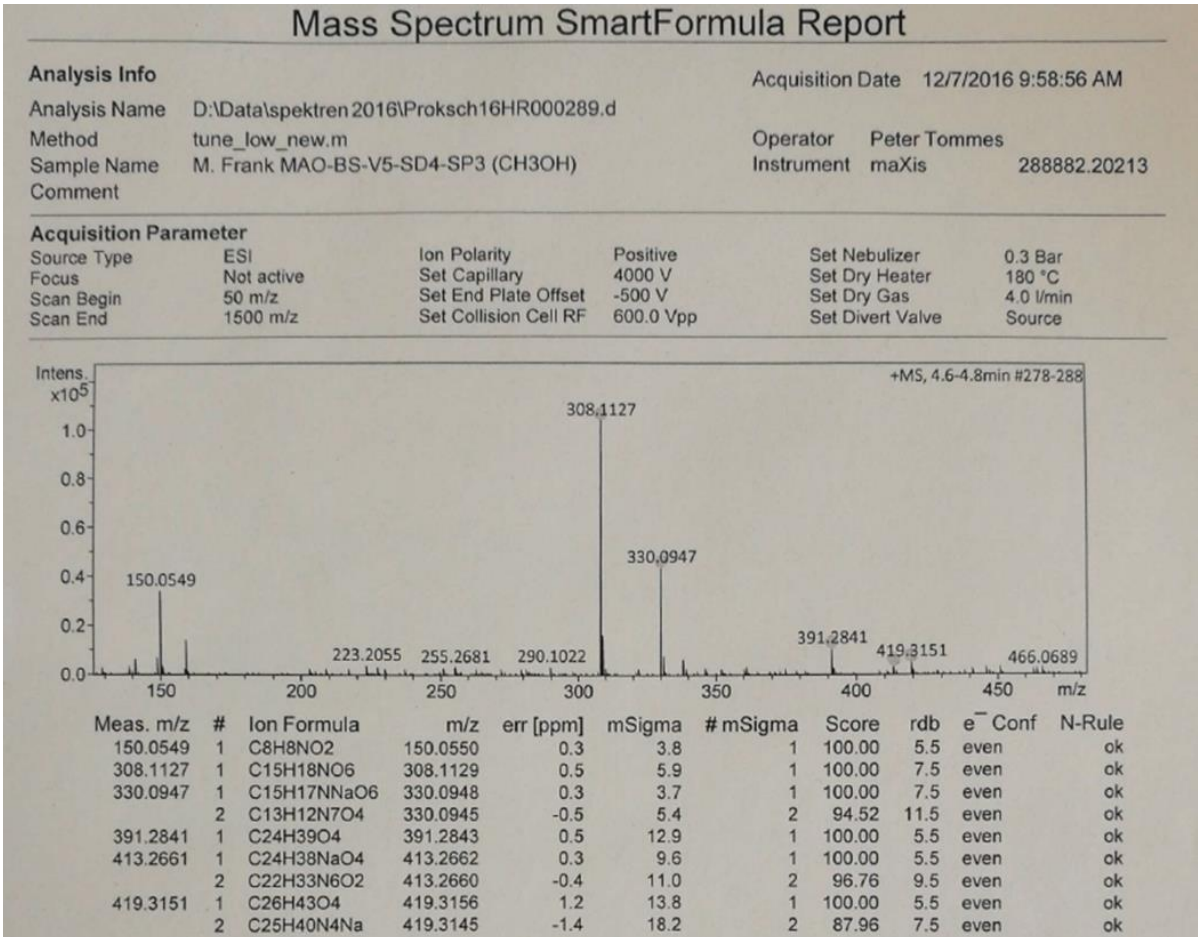

Mass Spectrum SmartFormula Report

Analysis Info

Acquisition Date 12/7/2016 9:58:56 AM

Analysis Name D:\Data\spektren 2016\Proksch16HR000289.d

Method tune_low_new.m

Operator Peter Tommes

Sample Name M. Frank MAO-BS-V5-SD4-SP3 (CH3OH)

Instrument maXis 288882.20213

Comment

Acquisition Parameter

Source Type	ESI	Ion Polarity	Positive	Set Nebulizer	0.3 Bar
Focus	Not active	Set Capillary	4000 V	Set Dry Heater	180 °C
Scan Begin	50 m/z	Set End Plate Offset	-500 V	Set Dry Gas	4.0 l/min
Scan End	1500 m/z	Set Collision Cell RF	600.0 Vpp	Set Divert Valve	Source

Meas. m/z	#	Ion Formula	m/z	err [ppm]	mSigma	# mSigma	Score	rdb	e⁻ Conf	N-Rule
150.0549	1	C8H8NO2	150.0550	0.3	3.8	1	100.00	5.5	even	ok
308.1127	1	C15H18NO6	308.1129	0.5	5.9	1	100.00	7.5	even	ok
330.0947	1	C15H17NNaO6	330.0948	0.3	3.7	1	100.00	7.5	even	ok
	2	C13H12N7O4	330.0945	-0.5	5.4	2	94.52	11.5	even	ok
391.2841	1	C24H39O4	391.2843	0.5	12.9	1	100.00	5.5	even	ok
413.2661	1	C24H38NaO4	413.2662	0.3	9.6	1	100.00	5.5	even	ok
	2	C22H33N6O2	413.2660	-0.4	11.0	2	96.76	9.5	even	ok
419.3151	1	C26H43O4	419.3156	1.2	13.8	1	100.00	5.5	even	ok
	2	C25H40N4Na	419.3145	-1.4	18.2	2	87.96	7.5	even	ok

Figure S17. ^{1}H NMR (600 MHz, MeOH-d_4) spectrum of compound **3**.

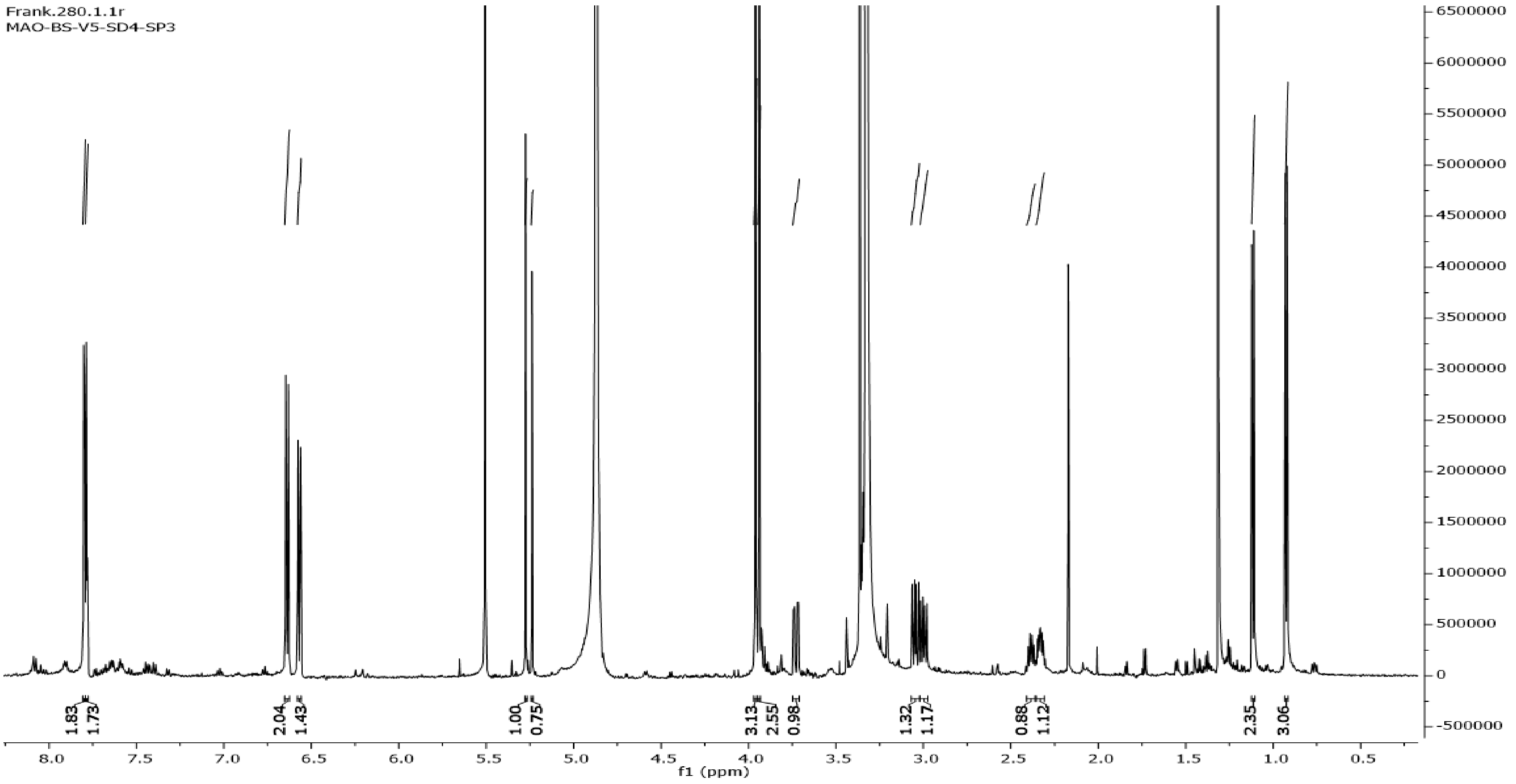

Figure S18. COSY (600 MHz, MeOH-d_4) spectrum of compound **3**.

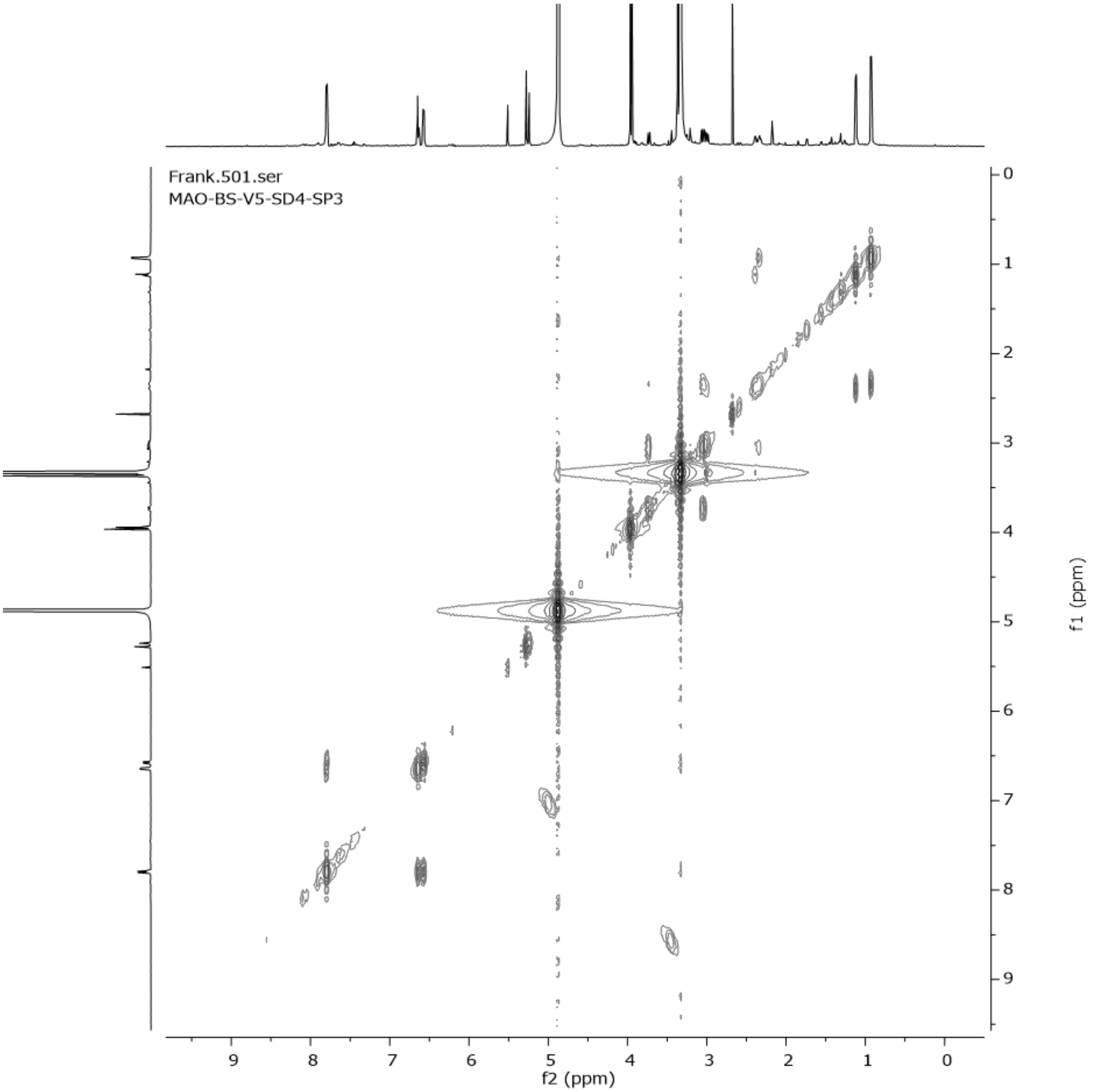

Figure S19. HSQC (600MHz/150 MHz, MeOH-d_4) spectrum of compound **3**.

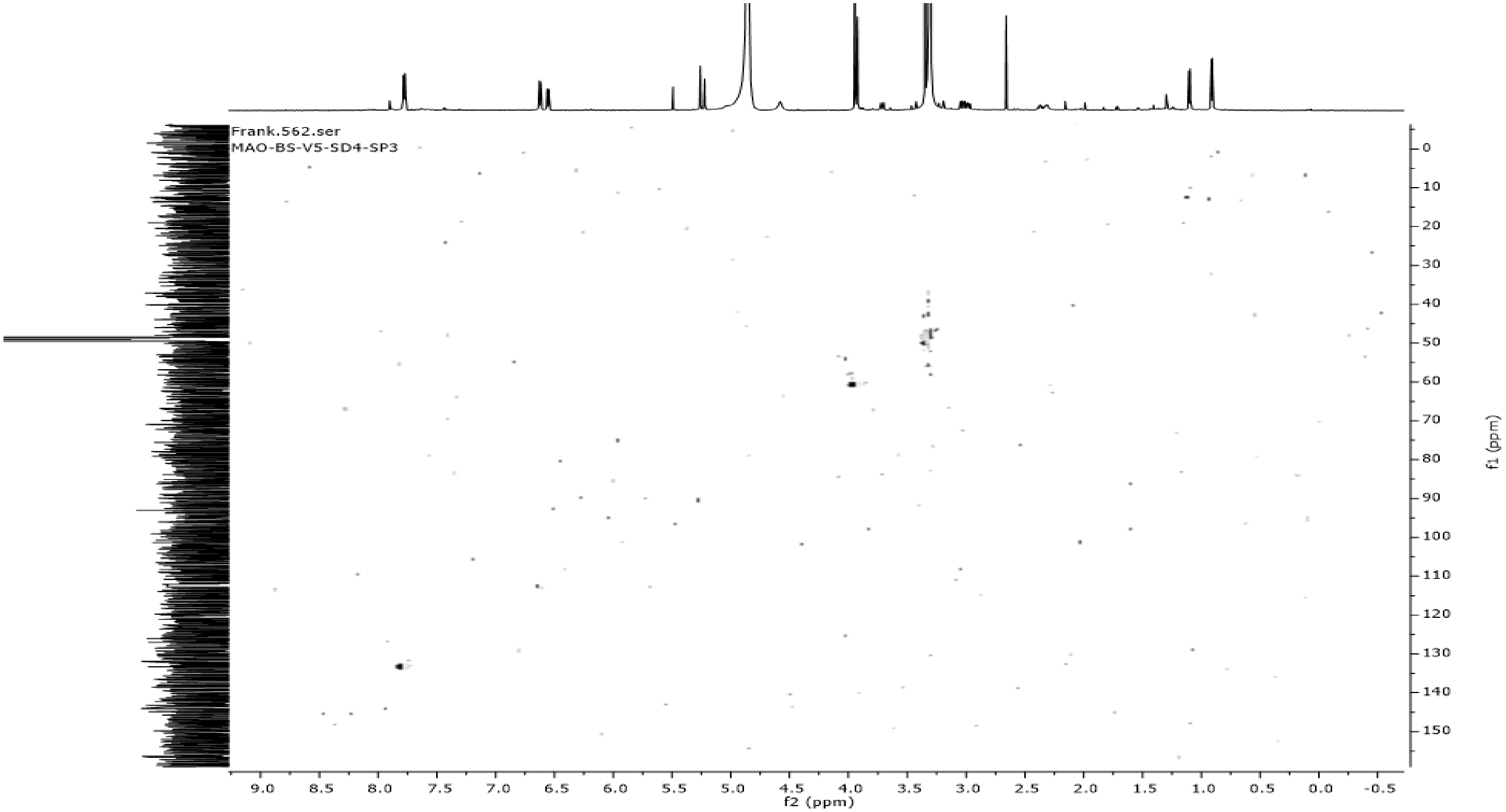

Figure S20. HMBC (600MHz/150 MHz, MeOH-d_4) spectrum of compound **3**.

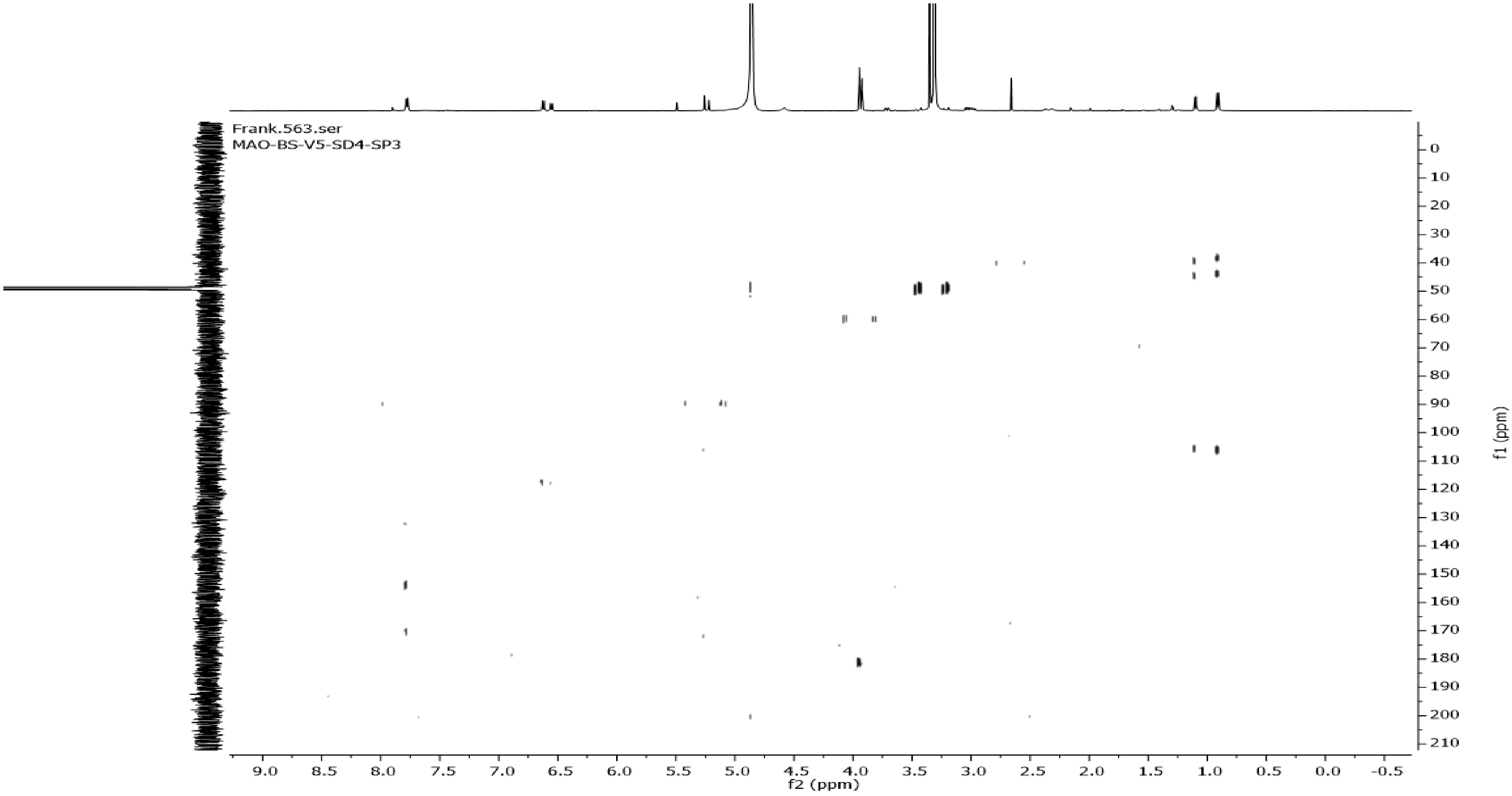

Figure S21. Chromatogram of C_4-Marfey's L-FDAA adduct of compound **1**.

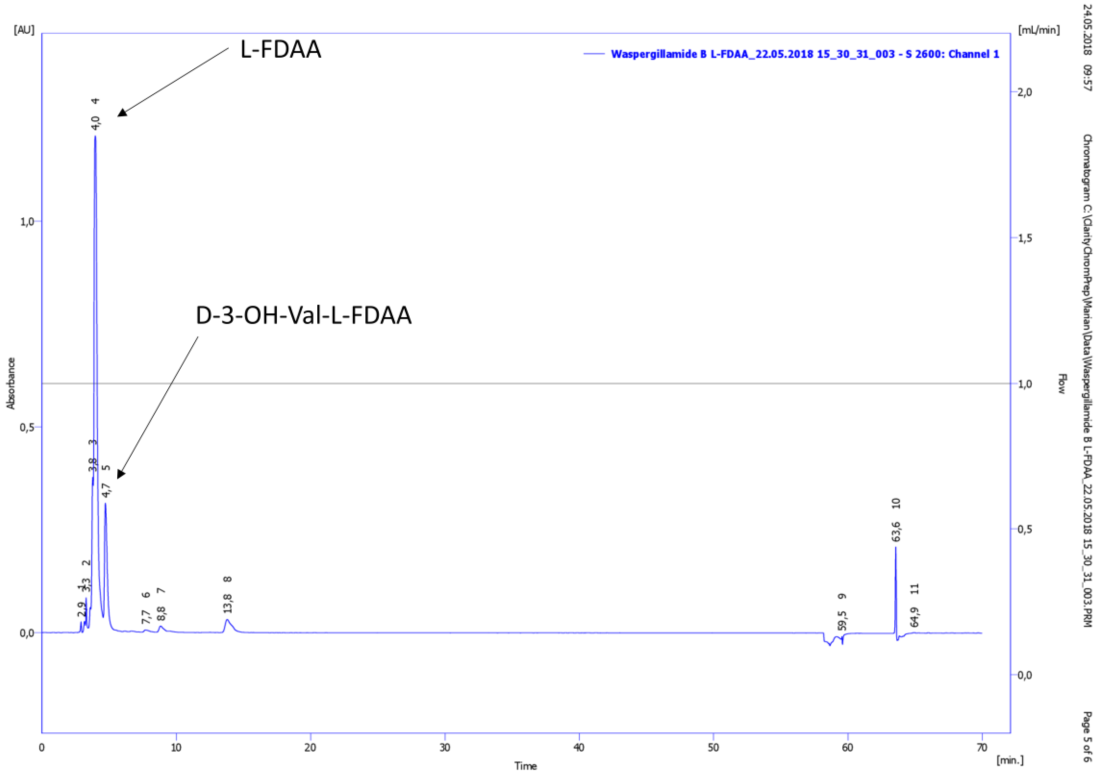

Figure S22. Chromatogram of C_4-Marfey's D-FDAA adduct of compound **1**.

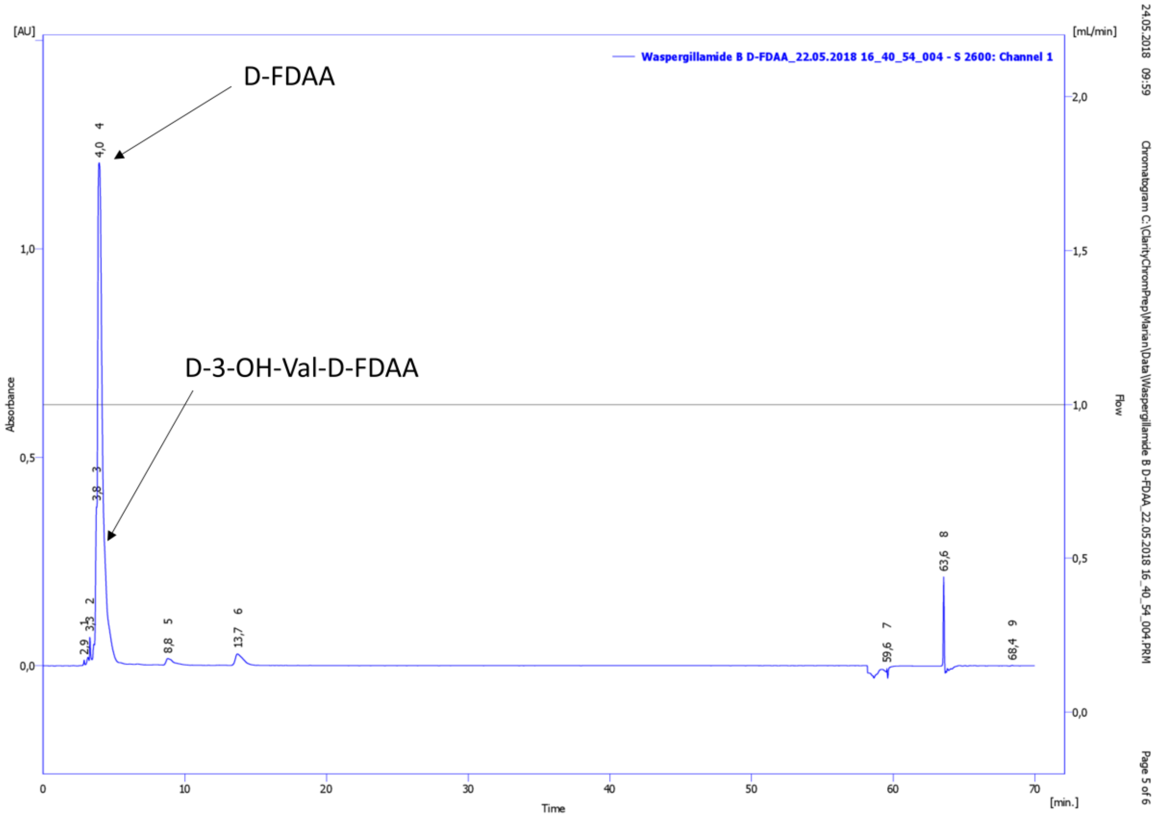

Supplement: Supplementary file 1 [file marinedrugs-17-00099-s001.pdf]
